# Acquired disorders of mitochondrial metabolism and dynamics in pulmonary arterial hypertension

**DOI:** 10.3389/fcell.2023.1105565

**Published:** 2023-02-02

**Authors:** Nolan M. Breault, Danchen Wu, Asish Dasgupta, Kuang-Hueih Chen, Stephen L. Archer

**Affiliations:** ^1^ Department of Medicine, Queen’s University, Kingston, ON, Canada; ^2^ Queen’s Cardiopulmonary Unit (QCPU), Translational Institute of Medicine (TIME), Department of Medicine, Queen’s University, Kingston, ON, Canada

**Keywords:** pyruvate dehydrogenase kinase (PDK), hypoxia-inducible factor 1-alpha (HIF-1α), dynamin-related protein 1 (DRP1), mitochondrial calcium uniporter complex (MCUC), pyruvate kinase (PK), Warburg metabolism

## Abstract

Pulmonary arterial hypertension (PAH) is an orphan disease of the cardiopulmonary unit that reflects an obstructive pulmonary vasculopathy and presents with hypertrophy, inflammation, fibrosis, and ultimately failure of the right ventricle (RVF). Despite treatment using pulmonary hypertension (PH)-targeted therapies, persistent functional impairment reduces the quality of life for people with PAH and death from RVF occurs in approximately 40% of patients within 5 years of diagnosis. PH-targeted therapeutics are primarily vasodilators and none, alone or in combination, are curative. This highlights a need to therapeutically explore molecular targets in other pathways that are involved in the pathogenesis of PAH. Several candidate pathways in PAH involve acquired mitochondrial dysfunction. These mitochondrial disorders include: 1) a shift in metabolism related to increased expression of pyruvate dehydrogenase kinase and pyruvate kinase, which together increase uncoupled glycolysis (Warburg metabolism); 2) disruption of oxygen-sensing related to increased expression of hypoxia-inducible factor 1α, resulting in a state of pseudohypoxia; 3) altered mitochondrial calcium homeostasis related to impaired function of the mitochondrial calcium uniporter complex, which elevates cytosolic calcium and reduces intramitochondrial calcium; and 4) abnormal mitochondrial dynamics related to increased expression of dynamin-related protein 1 and its binding partners, such as mitochondrial dynamics proteins of 49 kDa and 51 kDa, and depressed expression of mitofusin 2, resulting in increased mitotic fission. These acquired mitochondrial abnormalities increase proliferation and impair apoptosis in most pulmonary vascular cells (including endothelial cells, smooth muscle cells and fibroblasts). In the RV, Warburg metabolism and induction of glutaminolysis impairs bioenergetics and promotes hypokinesis, hypertrophy, and fibrosis. This review will explore our current knowledge of the causes and consequences of disordered mitochondrial function in PAH.

## 1 Introduction

Pulmonary hypertension (PH) is a heterogeneous family of cardiopulmonary diseases that are associated with symptoms of impaired exercise capacity and dyspnea. Regardless of the cause, a diagnosis of PH, defined simply as a mean pulmonary artery pressure (mPAP) > 20 mmHg at rest, increases mortality (Humbert et al., 2022). The sixth World Symposium on Pulmonary Hypertension organizes PH into 5 overarching groups, based on some degree of shared pathophysiology and therapeutic responses within the group (Simonneau et al., 2019). This review will focus on PAH, a subset of the PH syndromes contained within Group 1 PH.

PAH is a panvasculopathy, affecting all three unique vascular layers in the pulmonary arteries (PAs), including the tunica intima, media, and adventitia. This adverse vascular remodeling leads to vascular thickening and stiffening with a loss of compliance as well as a reduction in the cross-sectional area of the vascular bed. Hyperproliferation of pulmonary arterial endothelial cells (PAEC) and smooth muscle cells (PASMC) occurs in the intima and media, respectively. Fibroblasts also exhibit a hyperproliferative, fibrogenic phenotype in PAH, which has tissue-specific implications for the pulmonary vasculature *versus* the right ventricle (RV). Proliferation and enhanced collagen production by pulmonary arterial fibroblasts (PAfib) contribute to vascular remodeling and arterial stiffening, obstructing blood flow from the RV and promoting RV hypertrophy (RVH) (Bertero et al., 2015). Hyperproliferative fibroblasts in the RV also make excessive amounts of collagen, which increases ventricular stiffness and elevates RV diastolic pressures, a precursor to RVF (Tian et al., 2018; Tian et al., 2020a). Physiological RVH is adaptive, with increased cardiomyocyte size and the parallel organization of sarcomeres helping to reduce RV wall stress that is increased by elevated afterload due to pulmonary vascular disease. However, chronic RV pressure elevation in PAH often triggers a transition to pathological RVH, in which ischemia (due to vascular rarefaction and impaired perfusion of epicardial coronary arteries), as well as RV fibrosis (Tian et al., 2018), and inflammation (Al-Qazazi et al., 2022) leads to decompensation and RV failure (RVF), reviewed in Tian et al. (2020b).

The understanding of the mechanisms underlying PAH pathogenesis has evolved dramatically since the first antemortem hemodynamic and clinical description of the syndrome in 1951 (Dresdale et al., 1951). With this understanding has come improvements in therapy. Current treatments largely enhance vasodilatation by targeting pathways in PAEC, augmenting the nitric oxide-cyclic guanosine monophosphate (cGMP)-soluble guanylate cyclase pathway, the prostacyclin pathway, or inhibiting endothelin-1 signaling. While this approach does improve clinical outcomes, ∼90% of patients with PAH do not respond to acute vasodilators (defined as a failure to reduce mPAP by 20% to an mPAP <40 mmHg following acute vasodilator challenge during right heart catheterization). Thus, while current therapies often provide symptom relief, vasoconstriction is not the major cause of elevated mPAP in most patients with PAH (Humbert et al., 2006). Current PH-targeted drugs, alone or in combination, are therefore not curative and annual mortality rates in PAH patient populations remain above 10%, suggesting that new therapeutic targets are needed (Hoeper et al., 2017). Many PAH-inducing molecular pathways have been identified that may prove fruitful to this end. These include genetic targets, such as bone morphogenic protein receptor 2 (BMPR2) (Deng et al., 2000; International et al., 2000) and its ligands, inflammatory targets, such as the NLR family pyrin domain containing 3 (NLRP3) inflammasome and related cytokines (interleukin-6 and interleukin-1b) (Prins et al., 2018; Al-Qazazi et al., 2022), antiapoptotic pathways, such as those involving survivin (McMurtry et al., 2005) and DNA damage repair (Meloche et al., 2014), epigenetic pathways, such as those that increase DNA methylation (Archer et al., 2010a; Potus et al., 2020), transcription factor pathways involving hypoxia-inducible factor 1α (HIF-1α), nuclear factor of activated T Cells (NFAT) (Bonnet et al., 2007), signal transducer and activator of transcription 3 (STAT3) (Paulin et al., 2011) and angiogenic pathways involving vascular endothelial growth factor (VEGF) (Voelkel and Gomez-Arroyo, 2014), reviewed in (Thenappan et al., 2018).

However, our current research, and this review article, focus on a different frontier which identifies PAH as a syndrome that is partially due to acquired mitochondrial dysfunction, occurring downstream of multiple genetic and epigenetic abnormalities in both patients and animal models (Archer et al., 2008; Archer et al., 2010b). Owing to the central and multifaceted role of mitochondria in the regulation of cell metabolism, cell cycle progression, and apoptosis, mitochondrial dysfunction has been tied to a growing number of hallmark disease traits in PAH. These traits include uncoupled, aerobic glycolysis which is also called Warburg metabolism. Warburg metabolism permits hyperproliferation and apoptosis resistance in pulmonary vascular cells (Caruso et al., 2017). In PAH, there is also the induction of glutaminolysis in the RV (which promotes hypertrophy, hypokinesis and RVF) (Piao et al., 2013a), and inhibition of fatty acid oxidation (which promotes insulin resistance and cardiac steatosis) (Pugh et al., 2011; Brittain et al., 2016). In addition to metabolic remodeling, in the pulmonary circulation and RV, there is also an imbalance in mitochondrial dynamics, in that mitochondrial fission (division) is increased while mitochondrial fusion (joining) is impaired. In proliferating pulmonary vascular cells, mitochondrial fission permits accelerated cell cycle progression and impairs apoptosis. In the RV, increased mitochondrial fission has a different consequence, promoting the production of reactive oxygen species (ROS) and RV dysfunction. Like Warburg metabolism, abnormal mitochondrial dynamics offers new potential therapeutic targets for PAH (Marsboom et al., 2012a; Ryan et al., 2013; Chen et al., 2018). This mitochondria-centric view of PAH explains many of the pathologic processes seen in the pulmonary vasculature and RV in the disease. This review will explore our current knowledge pertaining to the function of mitochondria as master regulators of metabolism and cell division in PAH, revealing their potentially pivotal role in this devastating disease.

## 2 Mitochondrial metabolic dysfunction in PAH

### 2.1 Warburg metabolic shift

**FIGURE 1:**
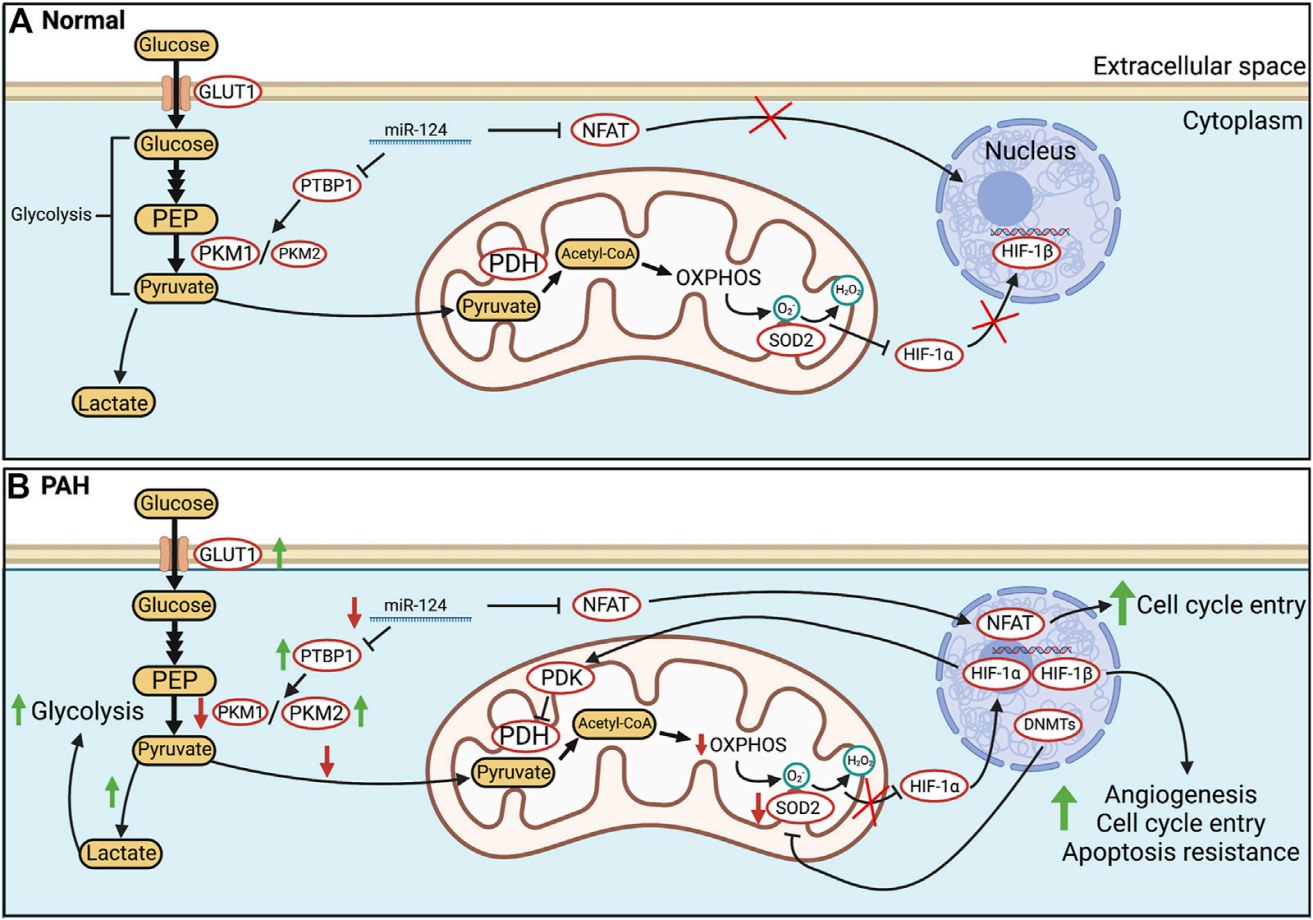
Glucose metabolism in health and PAH. **(A)**. In healthy PASMC, glycolytically-sourced pyruvate is transported to mitochondria and converted into acetyl-CoA for use in oxidative phosphorylation (OXPHOS). In normoxic PASMC, HIF-1α is inactivated *via* a basal production of mitochondria-derived H_2_O_2_. **(B)**. In human PAH and experimental PH, there is an epigenetic suppression of SOD2 and a related decrease in H_2_O_2_ production which activates HIF-1α, despite normoxic conditions. Transcriptional activation (activation of NFAT and HIF-1α) and epigenetic alterations (decreases in miR-124, and activation of DNA methyltransferases) occur in PAH PASMC. Decreases in miR-124 levels increase transcription of PTBP1 which increases the PKM2/PKM1 ratio, favouring glycolysis. In addition, HIF-1α increases the transcription of PDK which inhibits PDH. The net effects are enhanced uncoupled glycolysis and suppressed OXPHOS. These metabolic changes induce a proliferative, apoptosis-resistant state which, because of similarities to the PASMC’s phenotype upon exposure to environmental hypoxia, we refer to as a “pseudohypoxic state”. Abbreviations: DNMTs—DNA methyltransferases; GLUT1—Glucose transporter 1; HIF-1*α/β*—Hypoxia-inducible factor 1 α/β; PDH—Pyruvate dehydrogenase; OXPHOS—Oxidative phosphorylation; NFAT—Nuclear factor of activated T Cells; PASMC—Pulmonary artery smooth muscle cell; PDK—Pyruvate dehydrogenase kinase; PKM1/2—Pyruvate kinase muscle isoform 1/2; PTBP1—Polypyrimidine tract-binding protein 1; SOD2—Superoxide dismutase 2.

One of the central tenets of cellular energetics is the coupling of cytosolic glycolysis, which generates pyruvate, to the Krebs cycle in mitochondria. The Krebs cycle uses pyruvate as a substrate and produces reduced nicotinamide adenine dinucleotide (NADH) and reduced flavin adenine dinucleotide (FADH_2_), which are donors that provide electrons to the mitochondrial electron transport chain (ETC). The flow of electrons down the ETC towards molecular oxygen provides the electromotive potential that drives adenosine triphosphate (ATP) synthase and leads to the production of ATP (Figure 1) (Mitchell, 1961). In the RV and PAs of PAH patients and animals with experimental PAH, there is an increase in uncoupled glycolysis and often a depression of mitochondrial glucose oxidation (GO) (Piao et al., 2010; Caruso et al., 2017; Michelakis et al., 2017; Tian et al., 2020a). This metabolic hallmark of hyperproliferative diseases was first described by Otto Warburg in cancer cells (Warburg, 1956). When Warburg metabolism is operating, glycolysis terminates with the production of lactate in the cytosol instead of generating the pyruvate that is normally transported into the mitochondrial matrix by the pyruvate transporter. A metabolic reliance on uncoupled glycolysis reduces the synthesis of acetyl-CoA and downstream reducing equivalents, like NADH. Glycolysis alone generates only 2 moles of ATP/mole of glucose, whereas glycolysis coupled to oxidative phosphorylation (OXPHOS) generates an additional 36 moles of ATP, for a total of 38 moles. In addition to its inefficiency in generating ATP, glycolysis, when uncoupled, leads to lactic acidosis, which has unfavorable effects on cardiac function. While adaptive in hypoxic conditions, prolonged uncoupling of glycolysis from OXPHOS under normoxic conditions leads to a marked increase in glucose uptake (Marsboom et al., 2012b) and accelerated glycolysis, in a homeostatic effort to maintain ATP production. Although basal ATP levels are often preserved, Warburg metabolism impairs the RV’s bioenergetic reserve capacity and, in the vasculature, circumvents key regulatory functions of mitochondria, including their role in coordinating mitochondrial and nuclear division (mitotic fission) and in regulating the quality control process of apoptosis. This leads to tissue-dependent abnormalities, ranging from RV hypokinesis (Piao et al., 2010) to enhanced proliferation of pulmonary vascular cells (Li et al., 2016). Increased intracellular glucose also contributes to RV dysfunction by promoting posttranslational modification of cardiac proteins through a process called O-glcNAcylation (Prisco et al., 2020). The hexosamine biosynthetic pathway converts glucose to uridine-diphosphate-*N*-acetylglucosamine, which modifies serines or threonines and thereby alters protein function. Excess O-glcNAcylation contributes to RV dysfunction in experimental models of PAH and intriguingly, increased hemoglobin A1C levels are associated with RV dysfunction in patients with PAH (Prisco et al., 2020). However, the direct link between elevated HgbA1c levels and increased protein O-glcNAcylation requires further investigation. O-GlcNAcylation is likely one of the multiple pathways involved in increased HgbA1c-related RV dysfunction.

Under conditions in which Warburg metabolism operates, energy homeostasis is maintained by upregulation of cellular glucose intake through glucose transporters (Glut) (Zhang et al., 1999). This increased glucose flux is the basis for cancer detection by ^18^FDG positron emission tomography (PET) scanning, and this technique, which measures the intracellular uptake of ^18^F-fluorodeoxyglucose, detects Warburg metabolism in the RV and lung vasculature in both human and experimental PAH (Oikawa et al., 2005; Marsboom et al., 2012b; Zhao et al., 2013; Osorio Trujillo et al., 2021). Indeed, accelerated rates of vascular cell proliferation and the apoptosis-resistant nature of pulmonary vascular cells in PAH have long drawn comparison to cancer. This *pseudo neoplastic* phenotype of PAH relates to several acquired, epigenetically regulated mitochondrial abnormalities including a) an upregulation of enzymes that promote Warburg metabolism (pyruvate dehydrogenase kinase (PDK) and pyruvate kinase muscle isoform 2 (PKM2) and b) dysregulation of large GTPases that regulate mitochondrial dynamics, namely an increase in mitochondrial fission due to activation of dynamin-related protein 1 (DRP1) and an inhibition of fusion, related to downregulation of mitofusin 2 (MFN2).

A principal feature of the Warburg shift is the enhanced inhibition of pyruvate dehydrogenase (PDH) by PDK. PDH is the major mitochondrial regulatory enzyme for OXPHOS, catalyzing the conversion of pyruvate derived from glycolysis into acetyl-CoA for subsequent use in the Krebs cycle. PDK exists in four isoforms in mammalian cells, PDK1-4, each with unique affinities to cause inhibitory phosphorylation of PDH at serines-232, -293, and−300 (Korotchkina and Patel, 1995). PDK isoforms display tissue heterogeneity in their expression, with PDK1, 2, and four being the most prevalent in the heart and vascular tissue (Bowker-Kinley et al., 1998). The PDK inhibitor, dichloroacetate, is a small molecule that inhibits all four PDK isoforms. Dichloroacetate reduces phosphorylation of PDH and improves GO in PAH PASMC *in vitro* (McMurtry et al., 2004), in rats with experimental PAH (Michelakis et al., 2002a) and PAH patients (Michelakis et al., 2017). Dichloroacetate facilitates regression of vascular obstruction and improves RV contractility in PAH by improving GO in both the pulmonary vasculature (Michelakis et al., 2017) and RV (Piao et al., 2013b). Dichloroacetate also has little effect in normal tissues because these tissues lack significant PDK activity and PDH is not inhibited.

Multiple PDK isoforms are upregulated in PAH (Yuan et al., 2016; Michelakis et al., 2017). Their upregulated expression and activity is tied, at least partly, to the epigenetic activity of DNA methyltransferases that partially silence the redox regulatory gene *SOD2*, which encodes superoxide dismutase 2 (Archer et al., 2010a). Reduced SOD2 expression decreases the conversion of mitochondrial superoxide radicals into hydrogen peroxide (H_2_O_2_) and creates a reductive redox shift that activates HIF-1α, despite the presence of abundant oxygen (normoxia). HIF-1α′s activity is suppressed in normoxia by the activity of oxygen-sensitive prolyl hydroxylases, which mark it for ubiquitination by the von Hippel-Lindau protein, leading to rapid proteasomal degradation (Salceda and Caro, 1997). In hypoxia, prolyl hydroxylases are inhibited and HIF-1α becomes stabilized and translocates from the cytoplasm to the nucleus, where it binds HIF-1β and acts as a master regulator of hundreds of genes involved in adaptation to hypoxia (Manalo et al., 2005). HIF-1α increases the expression of genes related to angiogenesis, glycolysis, and cell cycle entry through binding to hypoxia-response elements (HRE) in the promoters of target genes (Lee et al., 2004). PDK1 and 2 expressions are directly upregulated by this HRE-mediated mechanism, while PDK4, lacking an HRE, is indirectly regulated by several mechanisms including HIF-1α-induced activation of estrogen-related-receptor γ expression, which exerts its own transcriptional control over PDK4 (Lee et al., 2012). Mice in which HIF-1α is knocked out are protected from PH induced by chronic exposure to environmental hypoxia (Yu et al., 2008). In the RV, PDK4 is transcriptionally regulated by FOXO1, rather than HIF-1α, and treatment with dichloroacetate in fawn-hooded rats reduces both FOXO1 and PDK4 expression (Piao et al., 2013b). Thus, there is heterogeneity in the regulation of PDK isoform expression, creating opportunities for tissue-specific therapy development. Interestingly, new evidence in the Sugen5416/hypoxia rat model of PAH (Su/Hx-PAH) also suggests the pentose phosphate pathway (PPP), which produces antioxidants and purines, also promotes a pro-proliferative gene program in vascular cells. PPP flux is enhanced by the increased expression of the rate-limiting enzyme, glucose-6-phosphate-dehydrogenase. This enhances NADPH production in favour of apoptosis resistance and supports DNA synthesis in favour of cell proliferation (Chettimada et al., 2015). In Su/Hx-PAH PASMCs, activation of the PPP also stabilizes HIF-1α and another transcription factor, specificity protein 1, which upregulates cyclin D to further accelerate cell cycle progression (Chettimada et al., 2015).

Another epigenetic mechanism by which Warburg metabolism and the hyperproliferative cell behavior is established in PAH relates to altered expression of microRNAs (miR), including miR-124. Reduced miR expression leads to increased survival of its target mRNAs and often increases expression of the encoded protein. Decreased expression of miR-124 has been implicated in the onset and metastasis of numerous cancers (Sun et al., 2012; Zhang et al., 2013). In PAH, decreased miR-124 is linked to increased glycolytic flux and proliferation in PAEC (Caruso et al., 2017), PASMC (Kang et al., 2013), and PAfib (Wang et al., 2014). In PAfib, downregulation of miR-124, secondary to decreased BMPR2 expression, increases the expression of the splicing factor, polypyrimidine tract-binding protein (PTBP1) (Caruso et al., 2017). PTBP1 enhances aerobic glycolysis by promoting alternative splicing of the gene encoding the terminal enzyme in glycolysis, pyruvate kinase (PK), which has four isoforms, M1, M2, L, and R (Prakasam et al., 2019; Zhu et al., 2020). L and R are primarily expressed in the liver and red blood cells, respectively, while M1 is the dominant isoform in most adult tissues (Prakasam et al., 2019). PKM2 is expressed in fetal cells and in rapidly proliferating cells and is upregulated in many malignant tumors (Zahra et al., 2020). miR-124 downregulation increases PTBP1 expression, favoring transcription of the PKM2 isoform (Caruso et al., 2017). Upregulated PKM2 expression in PAfib is tied to enhanced VEGF signaling (Luo et al., 2011), though the precise mechanisms connecting vascular insult to the Warburg shift and subsequent obliterative proliferation are unknown. This PKM2 predominance in PAH increases lactate production by 2-fold in idiopathic and heritable PAH while suppressing oxidative mitochondrial metabolism through a 3.5-fold increase in PDK1 and PDK2 expression (Caruso et al., 2017). In cancer cells and cardiomyocytes of group 2 PH, PKM2 is also a transcriptional co-activator for HIF-1α, constituting a positive feedback loop that favors a reliance on glycolysis (Luo et al., 2011). Decreased miR-124 also induces a downstream activation of NFAT in PASMC, leading to its translocation to the nucleus and induction of cell cycle entry that supports PASMC proliferation and adverse remodeling of pulmonary arteries (Bonnet et al., 2007). NFAT is a calcineurin-regulated transcription factor and its activity is increased in both chronic hypoxia PH (de Frutos et al., 2007) and monocrotaline models of PAH (MCT-PAH) (Figure 1) (Bonnet et al., 2007). NFAT activation decreases potassium ion efflux from PASMC, related to a downregulation of Kv1.5 channel expression (Bonnet et al., 2007). This Kv channel downregulation depolarizes PASMC membrane potential, which activates voltage-gated L-type calcium channels (Ca_L_), increases cytosolic calcium and induces SMC contraction. These ion channel signaling abnormalities contribute to elevated pulmonary vascular resistance and pressure, reviewed in Weir and Archer (1995). NFAT activation also inhibits apoptosis by enhancing bcl-2 expression, preventing localization of the pro-apoptotic factors Bax and Bak at the outer mitochondrial membrane (OMM) (Bonnet et al., 2007). NFAT’s contribution to PAH is thus mediated through both mitochondria-dependent and independent pathways, ultimately promoting both vasoconstriction and vascular obstruction. Small interfering RNA (siRNA) targeting NFAT reduces PASMC migration and proliferation while simultaneously restoring the sensitivity of PASMC to apoptosis in MCT-PAH (He et al., 2018). siNFAT mimics the effects of augmenting miR-124, suppressing NFAT-initiated cell division and contractility (Kang et al., 2013). miR-124’s targeting of PTBP1 and calmodulin-binding transcription activator 1 in PASMC further link its activity to the regulation of the cell cycle and mitochondrial metabolism (Kang et al., 2013). A similar breadth of control is observed in PAfib, where miR-124 plays a role in regulating inflammation, migration, and proliferation *via* PTBP1, NOTCH1, and HIF-2α (Wang et al., 2014). Therapeutic overexpression of miR-124 induces a growth arrest at the G1/S cell cycle checkpoint in PAfib of IPAH patients through direct interaction with the 3′-UTR of PTBP1 mRNA transcripts. This miR-mRNA interaction in PAfib, downregulating PTBP1 translation, is necessary for the control of downstream growth pathways and is epigenetically modulated by histone deacetylases (HDACs), which can be therapeutically targeted *via* HDAC inhibitors, such as apicidin and OSU42 (Wang et al., 2014).

In RV cardiomyocytes, which are mitochondria-rich and typically rely on fatty acid oxidation (FAO) for their energy needs, a shift to aerobic glycolysis, related to the normoxic activation of HIF-1α and other transcription factors like FOXO1, leads to vascular pruning and ischemia, promoting RVF (Bogaard et al., 2009). Similar glycolytic changes driven by HIF-1α are also seen in the lung in PAH (Marsboom et al., 2012b). In PAEC, initial vascular injury through a combination of genetics, epigenetics and exposure to hypoxia, inflammation, and/or drugs, like anorexigens or amphetamines, precedes neointimal formation. In the early stages of PAH, likely before the disease is clinically apparent, there may be a phase of excessive PAEC apoptosis; although by the time the disease is established, apoptosis resistance appears to predominate (Evans et al., 2021). Similar to the dysfunction seen in endothelial cells, PASMC and PAfib also attain a hyperproliferative, apoptosis-resistant phenotype that contributes to the pathophysiology of PAH by promoting medial hypertrophy and adventitial fibrosis (He et al., 2018). The timing of onset and the importance of Warburg metabolism in PAH likely varies amongst cell types and between patients with different etiologies for their PAH syndrome. However, as PAH progresses, each vascular layer in the pulmonary arteries—intima, media, and adventitia, becomes affected by a glycolytic mitochondrial metabolic phenotype, as are cardiomyocytes and fibroblasts in the RV. Indeed, our own studies show that ^18^FDG signals begin to increase in the lung early in the evolution of MCT-PAH when the hemodynamic abnormalities are mild (Marsboom et al., 2012b).

### 2.2 ROS production and oxygen sensing

In cancer, Warburg metabolism allows a partial avoidance of apoptosis by circumventing mitochondria-dependent apoptosis pathways and altering the production of reactive oxygen species (ROS). Superoxide anions, produced by electron leak at complexes I and III of the ETC during OXPHOS, are highly reactive with DNA and proteins but have a very limited diffusion radius. Mitochondria use SOD2 to convert superoxide to the less toxic and more stable and diffusible molecule, H_2_O_2_. Depending on the amount of H_2_O_2_ generated, this ROS can serve as a physiologic signaling molecule that regulates ion channels and enzymes by redox regulation of key sulfhydryl groups (as is the case in oxygen sensing) (Dunham-Snary et al., 2019) or cause pathology (as in RV ischemia-reperfusion injury) (Wu et al., 2020). H_2_O_2_ can initiate apoptosis by activating the tumor suppressor p53, which is normally repressed through binding by mouse double minute 2 (Wu et al., 1993). If a severe stressor is encountered in otherwise healthy cells, such as an abundance of ROS constituting oxidative stress, DNA damage, heat shock, or hypoxia, p53 may downregulate the transcription of survival proteins such as bcl-2 and survivin while upregulating pro-apoptotic factors, like Bax and Bak (Miyashita and Reed, 1995). Conversely, survivin is upregulated in both human and experimental PAH (McMurtry et al., 2005).

Cell signaling pathways that typically function as compensatory mechanisms in response to chronic hypoxia produce the same phenotype when triggered by acquired mitochondrial dysfunction and impaired oxygen sensing in PAH. For example, epigenetic methylation and downregulation of SOD2 by DNA methyltransferases in PAH (in the fawn hooded rat model of PAH) reduces H_2_O_2_-mediated signaling, mimicking the redox state seen in chronic hypoxia and eliciting PH that is characterized by medial thickening of the small pulmonary arteries and RVH (Bonnet et al., 2006; Archer et al., 2010a). The fawn hooded rat, which spontaneously develops PAH in normoxia, thus behaves as if they were living in hypoxia, due to epigenetically mediated mitochondrial dysfunction. However, conflicting evidence exists regarding the role of oxidative stress in establishing the PAH phenotype. Antioxidant supplementation using the small molecule TEMPOL, a SOD mimetic, attenuates increases in right ventricular systolic pressure if used prophylactically in Su/Hx-PAH (Jernigan et al., 2017). However, TEMPOL treatment failed to reduce RVH and exacerbated medial hypertrophy and fibrosis in small PAs (Jernigan et al., 2017). Conversely, in the fawn-hooded rat model, SOD supplementation regressed the PAH phenotype and improved aerobic capacity (Archer et al., 2010a). A mitochondria-targeted antioxidant, mitoTEMPO, is able to eliminate the hypoxia-induced ROS increase in PAEC (Adesina et al., 2015). It also improved exercise-induced PH by reducing pulmonary vascular remodeling and improving RV function in a rat model of combined pre- and post-capillary PH (Triantafyllou et al., 2021). Thus, it remains controversial whether superoxide and H_2_O_2_ promote or repress PAH pathophysiology. In PAH patients, markers of oxidative stress are increased. These markers include urine (Cracowski et al., 2001) and plasma (Zhang et al., 2014) isoprostane indicative of lipid peroxidation and ROS-induced DNA damage (Federici et al., 2015). While there is little debate about the presence of abnormal mitochondrial-derived ROS levels in PAH, the mechanisms connecting oxidative stress to the malignant phenotype of vascular cells remain imperfectly understood.

Changes in mitochondrial ROS production, because of Warburg metabolism, may simultaneously impact cell survival and oxygen sensing in PASMC. PASMC are integral to the homeostatic oxygen sensing system (HOSS). The HOSS is a collection of specialized tissues which detect and respond to hypoxia by altering vascular tone, neurosecretion, or neural activation with a resulting improvement in systemic oxygen delivery, reviewed in (Weir et al., 2005). Components of the HOSS include PASMC, ductus arteriosus (DA) SMC (Michelakis et al., 2002b), and fetoplacental arteries (Hampl et al., 2002), as well as non-vascular components, such as the neuroepithelial bodies in the lung (Fu et al., 2002) and the carotid body type 1 cell (Fernandez-Aguera et al., 2015). In the systemic arteries and DA, hypoxia induces vasodilation, whereas in the pulmonary and fetoplacental arteries, hypoxia yields vasoconstriction. In most HOSS tissues, the oxygen sensor resides within the mitochondria (Weir et al., 2005).

**FIGURE 2:**
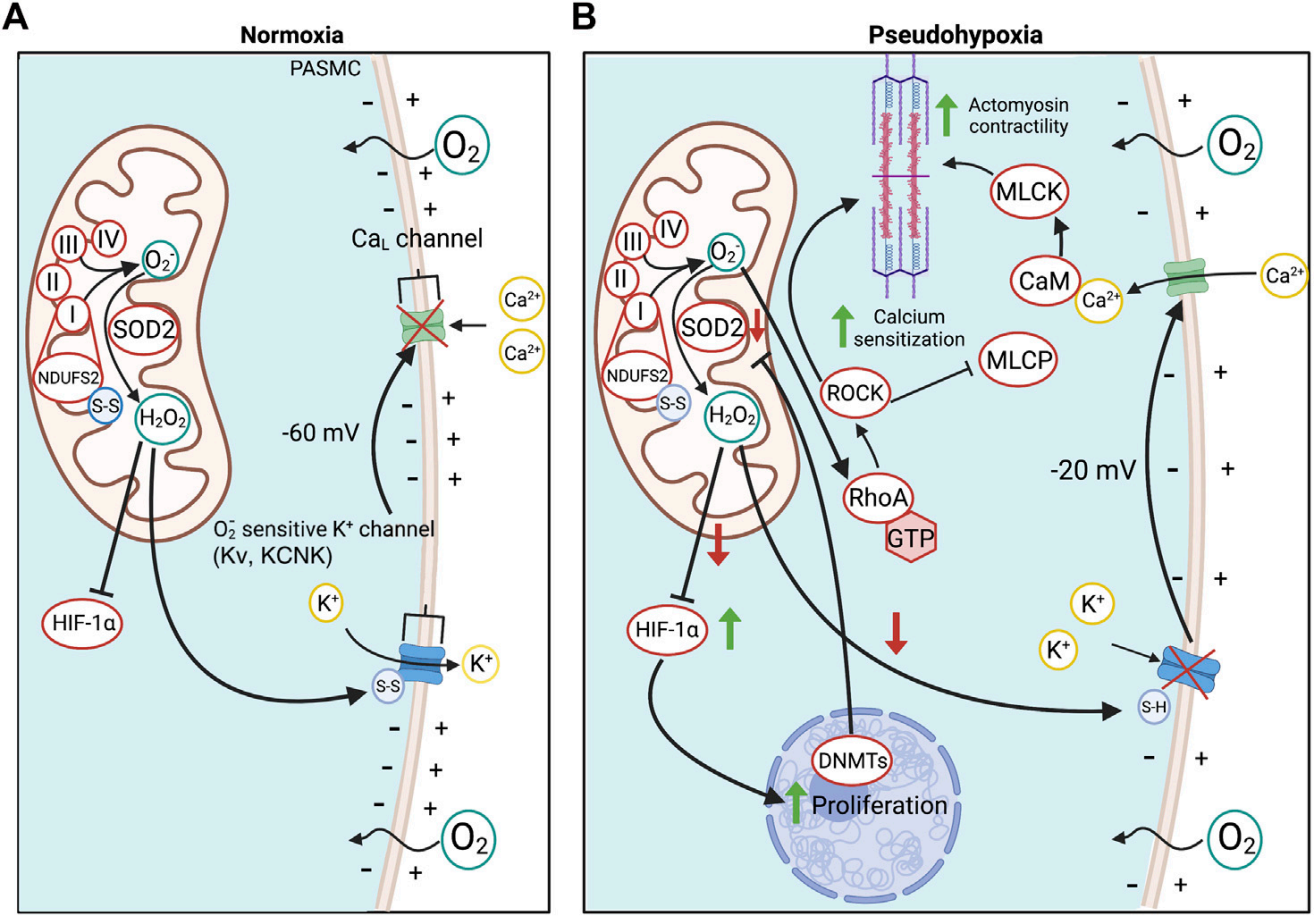
Oxygen sensing in PASMC in health and PAH. **(A)**. O_2_ detection in PASMC is enabled by the redox-sensitive NDUFS2 in ETC Complex I. NDUFS2 generates ROS during normoxia, creating a relatively oxidized state, whilst in hypoxia, NDUFS2 produces fewer radicals, creating a state of reduction. During normoxia, disulfide bridges in NDUFS2 are oxidized in support of ROS production and SOD2 converts O_2_^−^ to diffusible H_2_O_2_ for cytosolic export. H_2_O_2_ oxidizes redox-sensitive, voltage-gated, K^+^ channels, increasing their open state probability and maintaining tonic K^+^ egression, thereby hyperpolarizing the PASMC membrane potential and inactivating voltage-dependent calcium channels. This favours a state of normoxic relaxation. **(B)**. Epigenetic methylation of the *SOD2* gene in PAH by DNMTs reduces SOD2 expression and decreases normoxic production of H_2_O_2._ The resulting reduced redox state chemically reduces K^+^ channel disulfide bridges, inhibiting K^+^ channel opening and depolarizing the PASMC. As a result, voltage-dependent L-type Ca^2+^ channels (Ca_L_) are activated, increasing cytoplasmic Ca^2+^ which leads to vasoconstriction. Changes in redox state and cytosolic calcium also induce calcium/calmodulin (CaM)-dependent activation of myosin light chain kinase (MLCK), which creates a more sustained form of vasoconstriction. O_2_^−^ also activates ROCK through RhoA, promoting Ca^2+^ sensitization. Abbreviations: Ca_L_—L-type calcium channel; CaM—Calmodulin; DNMT—DNA methyltransferases; ETC—Electron transport chain; HIF-1α—Hypoxia-inducible factor 1α; KCNK4—Potassium channel subfamily K member four; Kv—Voltage-gated potassium channel; MCLK—Myosin light chain kinase; MLCP—Myosin light chain phosphatase; NDUFS2—NADH dehydrogenase [ubiquinone] iron-sulfur protein 2; RhoA - Ras homolog family member A; ROCK—Rho kinase; SOD2—Superoxide dismutase 2; ROS—Reactive oxygen species.

Hypoxic pulmonary vasoconstriction (HPV) is the lung’s homeostatic mechanism to match pulmonary blood flow to ventilation and thereby optimize oxygen uptake in conditions like pneumonia or atelectasis. Physiologic levels of alveolar hypoxia decrease ROS production, and the resulting decrease in diffusible H_2_O_2_ inhibits certain redox-sensitive potassium channels such as Kv1.5 and Kv2.1 (Archer et al., 1998; Platoshyn et al., 2001; Pozeg et al., 2003). The tonic egress of potassium down its intracellular/extracellular concentration gradient (140/5 mmol), *via* these channels, maintains the membrane potential of PASMC, setting the PASMC’s normoxic membrane potential at −60 mV. At these negative potentials, the open state probability of large conductance, voltage-dependent L-type calcium channels (Ca_L_) is low, which maintains low cytosolic calcium concentrations and, as a result, low resting tone in the normal pulmonary vasculature, reviewed in (Weir and Archer, 1995). During HPV, Kv channels close, leading to PASMC depolarization and an increase in the open-state probability of Ca_L_. This allows calcium to enter the cell and travel down a concentration gradient from 2 mmol in the extracellular space to ∼100 nmol in the cytosol (Dunham-Snary et al., 2017). Elevated cytosolic calcium causes vasoconstriction by activating the contractile apparatus through a calmodulin-myosin light chain kinase mechanism (Dabrowska et al., 1977), reinforced by the activation of rho kinase (ROCK), which inhibits myosin light chain phosphatase and enables sustained vasoconstriction (Uehata et al., 1997) (Figure 2).

Mitochondrial heterogeneity between tissues accounts for the observation that only certain tissues mount adaptive responses to hypoxia that increase systemic oxygen delivery. For example, in normal rats, PASMC mitochondria, but not renal arterial SMC, dynamically change their ROS production in response to physiologic levels of hypoxia, an attribute that appears to explain the unique localization of HPV to small PAs (Michelakis et al., 2002c). This PO_2_-dependent modulation of ROS production in PASMC is mediated in part by proteins within the ETC, such as NADH dehydrogenase [ubiquinone] iron-sulfur protein 2 (NDUFS2) of complex I (Dunham-Snary et al., 2019). In hypoxia, NDUFS2’s cysteine residues become reduced, which decreases the production of ROS (Dunham-Snary et al., 2019), creating a state of cytosolic reduction that inhibits K^+^ channels and elicits HPV, as reviewed in (Weir et al., 2005). NDUFS2 in ETC complex I also participates in mitochondrial oxygen sensing in the carotid body type 1 cell (Fernandez-Aguera et al., 2015).

Multiple aspects of the signaling pathway that mediates HPV are dysfunctional in PAH. For example, the normoxic activation of HIF-1α, also seen in cancer cells (Jun et al., 2017), creates a “pseudohypoxic state” that pathologically activates proglycolytic metabolism and promotes cell cycle entry. HIF-1α activation also reduces Kv channel expression and mice that are haploinsufficient for HIF-1α are protected from the effects of chronic hypoxia, manifesting less hypoxia-induced depolarization, less reduction of K^+^ current density, and less PASMC hypertrophy (Shimoda et al., 2001). In normoxic PAH PASMC, the membrane potential is depolarized to levels seen in normal PASMC upon exposure to acute hypoxia. The resulting elevation of intracellular Ca^2+^ concentrations is a stimulus for vasoconstriction. However, the sustained inhibition or downregulation of K^+^ channels also impairs apoptosis (Burg et al., 2008). This impairment of apoptosis relates in part to elevated cytosolic K^+^ concentrations which inhibit endogenous caspases and nucleases and can suppress mitochondrial cytochrome *c* release.

O_2_-sensitive Kv channels, such as Kv1.5, are downregulated in PASMC, both in preclinical PAH models (Michelakis et al., 2001; Bonnet et al., 2006) and in PASMC isolated from PAH patients (Yuan et al., 1998) due, in part, to upregulation of miR-22b, miR-138 and miR-222 (Babicheva et al., 2020). Evidence of the pathogenic importance of disordered O_2_-sensing pathways in PAH includes the observations that therapeutic overexpression of Kv1.5 (Michelakis et al., 2001; Brevnova et al., 2004; Bonnet et al., 2006) or dominant negative HIF-1α inhibition (Bonnet et al., 2006) restores PASMC membrane polarity and apoptosis *in vitro.* Dichloroacetate, an inhibitor of PDK that restores oxidative glucose metabolism in PAH PASMC, also restores Kv1.5 expression in animal models of PAH and regresses adverse pulmonary remodeling and improves hemodynamics (Bonnet et al., 2006). Similar phenomena are seen in the case of the pH-sensitive KCNK3 channel, which are two-pore-forming P domain (TASK-1) channels. KCNK3 channels also contribute to resting PASMC membrane potential through K^+^ efflux. KCNK3 activity is principally controlled by the protonation state of its histidine-98 residue (Morton et al., 2003). Hypoxia-associated acidosis and reduction of the H98 imidazole group inhibit outward potassium flow, while alkalosis activates the channel. Mutations in KCNK3 have been found in 3.2% of hereditary PAH cases and in 1.3% of patients with IPAH (Ma et al., 2013). KCNK3 mutations result in a loss of channel function, which contributes to PASMC depolarization and elevated vascular tone (Ma et al., 2013). Furthermore, rats with loss of function KCNK3 mutations develop the characteristic hyperproliferative cell phenotype seen in PAH and manifest pathologic activation of kinases like SRC and extracellular signal-regulated kinase (ERK) 1/2, as well as the normoxic activation of HIF-1α (Lambert et al., 2019).

Collectively, the dysregulation of these signaling pathways in PAH creates a state of *pseudohypoxia*, wherein cells of the pulmonary vasculature, despite normoxic conditions, manifest activation of HIF-1α and an acquired channelopathy which promotes pulmonary vascular constriction and remodeling typical of that seen after chronic hypoxia exposure. The precise order of events in the creation of the pseudohypoxic state remains unknown, though the number of altered mitochondrial signaling pathways in oxygen-sensing cells suggests the intimate involvement of these organelles.

### 2.3 Calcium flux

**FIGURE 3:**
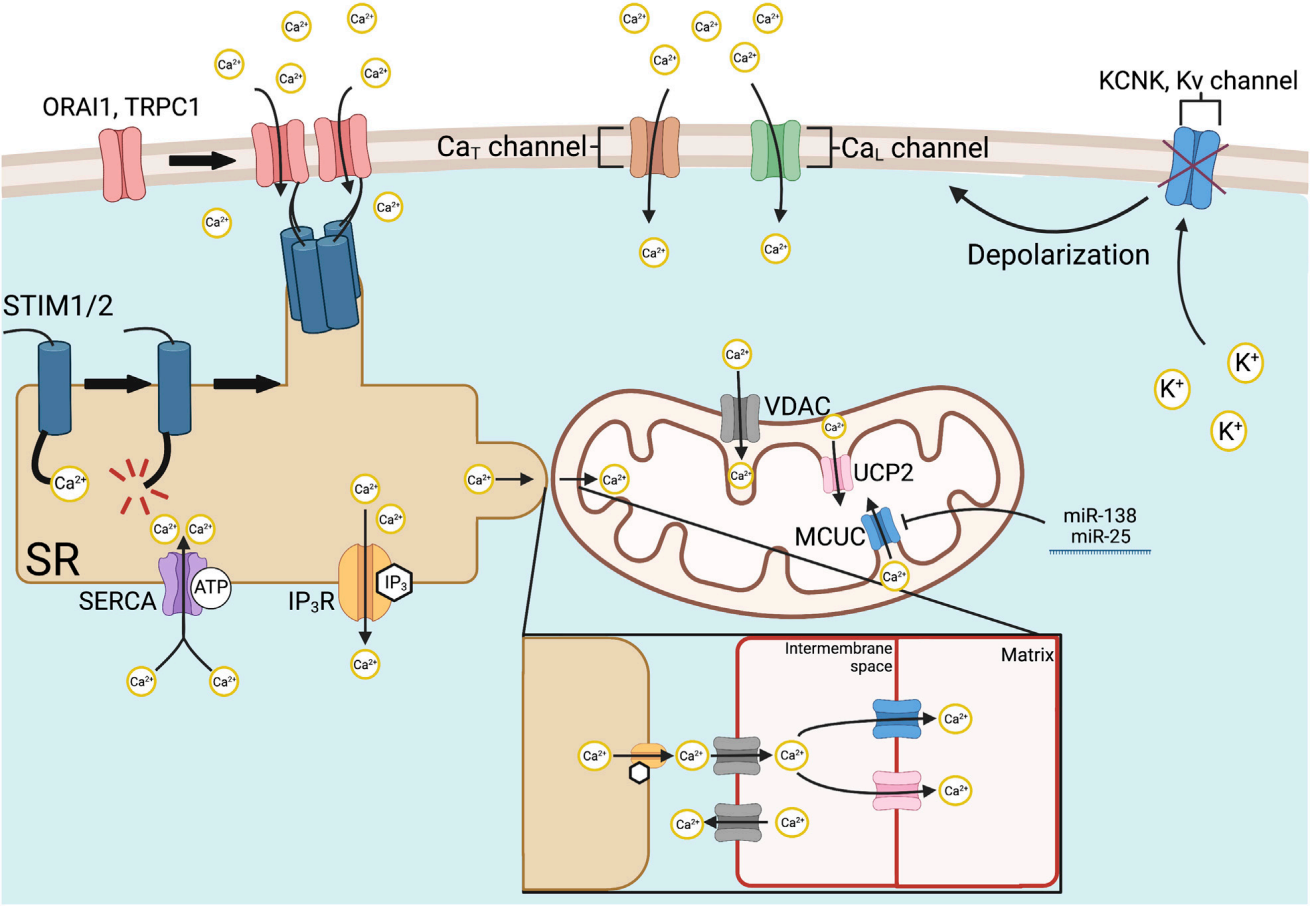
Mechanisms of intracellular Ca^2+^ regulation. Store-operated Ca^2+^ entry is initiated *via* SR-bound STIM1 and STIM2, which have Ca^2+^-sensitive EF hand domains. Ca^2+^ store depletion from the SR causes STIM1/2 to aggregate at SR-plasma membrane junctions and recruits ORAI1, TRPC1, and other Ca^2+^ channels by its STIM1 ORAI activating region or CRAC activating domain. Various Ca^2+^ channels allow organellar uptake, such as SERCA and release, such as IP_3_Rs, respectively. Mitochondrial Ca^2+^ uptake is mediated by the MCUC. The channel-forming subunit of the MCUC, MCU, is epigenetically downregulated in PAH by increased expression of miR-138 and miR-25. MCUC inhibition contributes to the Warburg effect by inactivating Ca^2+^-dependent enzymes that regulate OXPHOS within the mitochondria. In addition, reduced MCUC function increases cytosolic calcium, favoring vasoconstriction. Intramitochondrial calcium is also regulated, under certain conditions by the VDAC on the outer mitochondrial membrane, and UCP2, on the inner mitochondrial membrane. In PAH, voltage-gated Ca^2+^ entry is initiated by the inhibition and downregulated expression of redox-sensitive K^+^ channels, such as Kv1.5. The loss of tonic K+ efflux depolarizes the PASMC membrane potential, thereby activating L- and T-type Ca^2+^ channels and allowing influx of extracellular calcium down its 20,000/1 extracellular to intracellular concentration gradient. Abbreviations: Ca_L/T_—L/T-type calcium channel; IP_3_R—Inositol-1,4,5-triphosphate receptor; KCNK4—Potassium channel subfamily K member four; Kv—Voltage-gated potassium channel; MCUC—Mitochondrial calcium uniporter complex; OXPHOS—Oxidative phosphorylation; PAH—Pulmonary arterial hypertension; PASMC—Pulmonary artery smooth muscle cell; SERCA—Sarco (endo)plasmic reticulum calcium ATPase; SR—Sarcoplasmic reticulum; ORAI1—Calcium release-activated calcium channel protein 1; STIM1/2—Stromal interaction molecule 1/2; TRPC1—Transient receptor potential cation channel subfamily C member 1; UCP2—Uncoupling protein 2; VDAC—Voltage-dependent anion channel.

Calcium homeostasis, which is central to the regulation of mitochondrial metabolism, vascular tone and PASMC proliferation, is disordered in PAH. In addition to the important role of calcium influx *via* Ca_L_ channels, extracellular calcium entry in SMC occurs *via* store-operated calcium channels (SOCCs) (Figure 3). SOCCs are activated in response to depletion of intracellular calcium reservoirs in mitochondria, as well as the endoplasmic (ER) and sarcoplasmic reticulum (SR). Store-operated calcium entry (SOCE) is initiated by the action of STIM1, a transmembrane protein that is bound to the ER and SR. ER- and SR-specific calcium store levels are altered *via* the sarco (endo)plasmic reticulum calcium ATPase (SERCA) and inositol-1,4,5-triphosphate receptors (IP_3_R), which mediate calcium influx and release, respectively. SERCA activity is dependent on a variety of factors, including cellular pH and the presence of peptide effectors such as phospholamban. The consumption of ATP by SERCA leads to its phosphorylation and activation, allowing for the transport of two calcium ions from the cytosol into the SR lumen per ATP consumed (Inesi, 1987). Conversely, IP_3_R, which activates during receptor-operated calcium entry, serves to deplete intraluminal calcium. Detection of low calcium store levels is performed by the EF hand domain of STIM1, which initiates its translocation to ER/SR-plasma membrane junctions (Liou et al., 2005). Here, STIM1 uses its C-terminal STIM1 ORAI activating region, also known as the CRAC activation domain, to recruit calcium release-activated calcium channel protein 1 (ORAI1) (Park et al., 2009), transient receptor potential canonical 1 (TRPC1), and other calcium channels for SOCE (Yuan et al., 2009; Rodriguez-Moyano et al., 2013). Increases in this cellular signaling pathway are implicated in SMC proliferation. For example, treatment of aortic SMC with siRNA targeted towards STIM1, ORAI1, and TRPC1 reduces calcium influx and inhibits cell division (Vallot et al., 2000). Similarly, selective blocking of TRPC6 in a chronic hypoxia mouse model of PH, *via* intrapulmonary administration of BI-749327, regresses established PH (Jain et al., 2021). Daily treatment with BI-749327 over 2 weeks led to reductions in right ventricular systolic pressure, mPAP, and Fulton index of 47%, 47% and 53%, respectively, relative to PH-affected control animals (Jain et al., 2021). In mouse PASMC, 24-hour incubation with 50 µM BI-749327 also inhibits platelet-derived growth factor (PDGF)-mediated phosphorylation of mammalian target of rapamycin (mTOR) and AKT, suggesting that TRPC6 targeting may be a useful avenue for addressing vascular cell hyperproliferation and the decline in right ventricular function in PAH (Jain et al., 2021). Additional evidence of the relationship between cytoplasmic calcium levels and cell proliferation exists in the downregulation of SERCA in vascular SMC and endothelial cells. In studies using human umbilical vein cells and rodent vascular SMC, the loss of SERCA-mediated calcium uptake prevents cell cycle entry (Mountian et al., 1999; Vallot et al., 2000; Lipskaia et al., 2003). In SMC, SERCA is downregulated in response to growth factor activation, which connects mitogens, such as PDGF, and decreased SERCA activity, to the pathogenesis of IPAH (Vallot et al., 2000; Perros et al., 2008). SERCA2a is also downregulated in human PAH-affected PASMC, further contributing to the cytosolic calcium overload, and in MCT-PAH, adenovirus-mediated gene therapy of SERCA2a reduces mPAP and vascular hypertrophy (Hadri et al., 2013). Conversely, SERCA overexpression in both cultured human PASMC and the rat MCT-PAH has therapeutic benefits. SERCA overexpression increases nitric oxide synthase (NOS) expression in PAEC while improving pulmonary hemodynamics and reducing both RVH and RV fibrosis in MCT-PAH (Hadri et al., 2013).

Further revealing the connection of SOCE to the pathogenesis of PAH is the upregulation of STIM2 and the associated increase in resting cytosolic calcium levels in IPAH. STIM2 bears a high degree of structural homology to STIM1 and interacts with ORAI1 and other SOCCs commonly involved in STIM1 activity. IPAH PASMC has been shown to express 45% more STIM2 peptide and have a corresponding 35% increase in resting cytosolic calcium (Song et al., 2018). This increase in calcium is partially tied to STIM2, as healthy PASMC in which STIM2 is overexpressed recapitulate the calcium overload observed in IPAH PASMC (Song et al., 2018). However, STIM2’s lower affinity for calcium ions in its EF domain has led to the suggestion that it is more responsible for maintaining resting cytosolic calcium levels, while STIM1 controls calcium flux downstream of extracellular stimuli that elevate cytosolic calcium (Brandman et al., 2007). Enhanced STIM2 protein levels promote SMC proliferation and vascular remodeling by activating a variety of transcription factors, including NFAT, CREB, STAT3, and AKT, even in the absence of calcium store depletion (Song et al., 2018). Upregulation of STIM2 in PAH appears to be unique amongst ER and SR calcium sensors, as STIM1 levels are unchanged in IPAH, although the expression of ORAI1, TRPC1, and the anti-apoptotic protein Bcl-2 are also increased (Song et al., 2018). The underlying mechanism behind STIM2 dysregulation in PAH and the relative contribution of each SOCC mediator to the development of PAH requires further study. As such, the pathways that regulate store-operated calcium release represent a largely unexplored therapeutic target in PAH.

The role of voltage-gated calcium channels (Ca_v_1) in HPV and PAH, however, is well-established (Weir and Archer, 1995). In PASMC, the primary mediators of voltage-dependent calcium entry are voltage-activated Ca_L_ channels, which are inhibited by dihydropyridines, like nifedipine. Indeed, in the ∼10% of PAH patients who have a robust acute vasodilator response, therapy with nifedipine is quite effective (Sitbon et al., 2005; Montani et al., 2010). Ca_L_ requires substantial depolarization to be activated, as occurs with inhibition or downregulation of voltage-gated potassium channels. As previously discussed, Ca_L_ activity is regulated by the passive export of potassium through channels such as KCNK3, Kv1.5, and Kv2.1. However, calcium uptake and release by mitochondria, as well as by the ER and SR, also regulate Ca_L_ activity. Once activated, however, voltage-gated calcium channels can conduct sustained calcium currents.

In comparison to Ca_L_-based signaling, T-type calcium channel (Ca_T_) activity in the context of PAH is relatively unexplored, partly due to a historical lack of specific pharmacological inhibitors (Perez-Reyes, 2003; Kuo et al., 2011). Recent evidence suggests that Ca_T_ currents regulate PASMC cell cycle progression and apoptosis resistance in both IPAH (Sankhe et al., 2017) and Group 3 PH (Chevalier et al., 2014). Fine changes in cell membrane potential lead to small depolarizations that activate Ca_T_, and these channels, in turn, mediate transient calcium influx (Perez-Reyes, 2003). In IPAH PASMC, upregulation of Ca_T_ leads to a loss of protein phosphatase 2A (PP2A) activity by an unknown mechanism (Sankhe et al., 2017). This decrease in phosphatase function enhances the activity of ERK1/2-, AKT1-, and P38-mediated cell cycle progression in IPAH (Sankhe et al., 2017). Selective blockade of Ca_T_ (Cav3.1 and Cav3.2) by the small molecule TTA-A2 inhibits growth and metastasis in A549 lung cancer spheroids (Kumari et al., 2020), though its effects in PAH remain unknown. Gilbert et al. also discovered a vasodilatory role of Ca_T_ in PAEC, wherein NOS and Ca_T_ colocalization mediates localized increases in intracellular calcium, thereby increasing NOS activity (Gilbert et al., 2017). Further study of the role of Ca_T_ in PAH is important.

Mitochondria are another major intracellular calcium store. Mitochondrial calcium uptake is mainly accomplished through the action of the mitochondrial calcium uniporter complex (MCUC), located on the inner mitochondrial membrane (IMM). The MCUC is composed of the channel-forming proteins MCU (Baughman et al., 2011; De Stefani et al., 2011) and essential MCU regulator (EMRE) (Sancak et al., 2013). Other MCUC components are regulatory proteins, including mitochondrial calcium uptake 1 (MICU1) (Perocchi et al., 2010) and mitochondrial calcium uptake 2 (MICU2) (Plovanich et al., 2013). The MCU subunit forms a tetrameric pore between the intramembranous space and the mitochondrial matrix (Baradaran et al., 2018), allowing the influx of calcium ions into the matrix in support of aerobic metabolism through calcium’s role as a cofactor for both PDH phosphatase and various dehydrogenases that mediate the Krebs cycle. EMRE also plays a critical role in calcium import by controlling MCU pore opening *via* three primary sites of contact with MCU and its transmembrane helices, which regulate MCU’s open state (Van Keuren et al., 2020). In this way, mitochondrial calcium influx regulates OXPHOS either directly, such as *via* controlling isocitrate dehydrogenase or oxoglutarate dehydrogenase activation, or indirectly, by activating PDH phosphatase, which in turn activates PDH (Denton et al., 1980; McCormack and Denton, 1993). In PAH, MCU function is reduced, which results in a reduction in intramitochondrial calcium concentrations. Decreased MCUC function results in part from an epigenetic mechanism mediated by elevated expression of miR-138 and miR-25 (Hong et al., 2017). Compounding the downregulation of MCU, the MICU1 subunit of the MCUC, which acts as a negative regulator of MCU-mediated calcium import, is upregulated in PAH (Hong et al., 2017).

Further exacerbating the calcium imbalance caused by MCUC dysfunction in PAH is an increase in ROCK-mediated calcium sensitization of PAH PASMC. ROCK increases the contractility of myosin by inactivating myosin light chain phosphatase, permitting sustained vasoconstriction, independent of calcium influx. Thus, ROCK activation leads to calcium sensitization (sustained vasoconstriction, which is resistant to Ca_L_ inhibitors). The activity of the G protein RhoA and its downstream target ROCK are increased in both IPAH and animal PAH models, particularly because of serotonylation- (Guilluy et al., 2009) and superoxide-mediated (Resta et al., 2010) activation of RhoA which results in constitutive Ca^2+^ hypersensitivity. Vasoconstriction induced by this pathway is unresponsive to conventional vasodilators, though ROCK inhibitors, such as fasudil, can counteract constriction caused by calcium sensitization and are effective in treating clinical RVF in patients with severe pulmonary hypertension (Jiang et al., 2015).

### 2.4 Glutaminolysis

Upregulated glutaminolysis is a recently discovered mitochondrial metabolic abnormality in PAH (Piao et al., 2013a). Glutamine is the most abundant circulating amino acid and has diverse metabolic fates as a carbon and nitrogen donor. This feature underlies the importance of glutaminolysis in vital functions of actively proliferating or hypertrophying cells, supporting their need for increased membrane lipids and nucleic acids. In most adult tissues, glutaminolysis contributes little to metabolic homeostasis; however, proliferating cells, including those of the gastrointestinal tract and kidneys, derive a large fraction of anabolic molecules and energy from glutamine (Lacey and Wilmore, 1990).

Glutaminolysis is also important in the hypertrophied RV in PAH. Piao *et al.* demonstrated that RV cardiomyocytes from rats with MCT-PAH have *de novo* activation of glutamine metabolism which contributes to RVH and eventual RV failure. In MCT-PAH, the RV demonstrates ischemia (due to capillary rarefaction and reduced epicardial perfusion pressure in the right coronary artery) (Tian et al., 2020b) and has increased expression of the glutamine transporter SLC1A5 relative to the pulmonary artery-banded rat model, despite similar severity of RVH (Piao et al., 2013a). Increased SLC1A5 expression is also seen in the RV myocytes of patients with PAH (Piao et al., 2013a). Transcriptional upregulation of glutaminolysis appears to be mediated by the transcription factors c-Myc and Max, which are upregulated in the MCT RV (Piao et al., 2013a). In MCT-PAH, a six-fold increase in glutamine uptake was observed in the heart *ex vivo* using a dual isotope technique. *In vivo*, administration of an inhibitor of glutaminolysis, 6-diazo-5-oxo-L-norleucine (DON), reduced RVH and increased cardiac output (Piao et al., 2013a), suggesting the net effect of increased glutaminolysis in the PAH RV is maladaptive. Interestingly, inhibition of glutaminolysis also caused a reciprocal increase in GO (Piao et al., 2013a), suggesting a reciprocal relationship between these pathways, akin to the reciprocal relationship between GO and FAO, which is called the Randle cycle (Randle et al., 1963).

**FIGURE 4:**
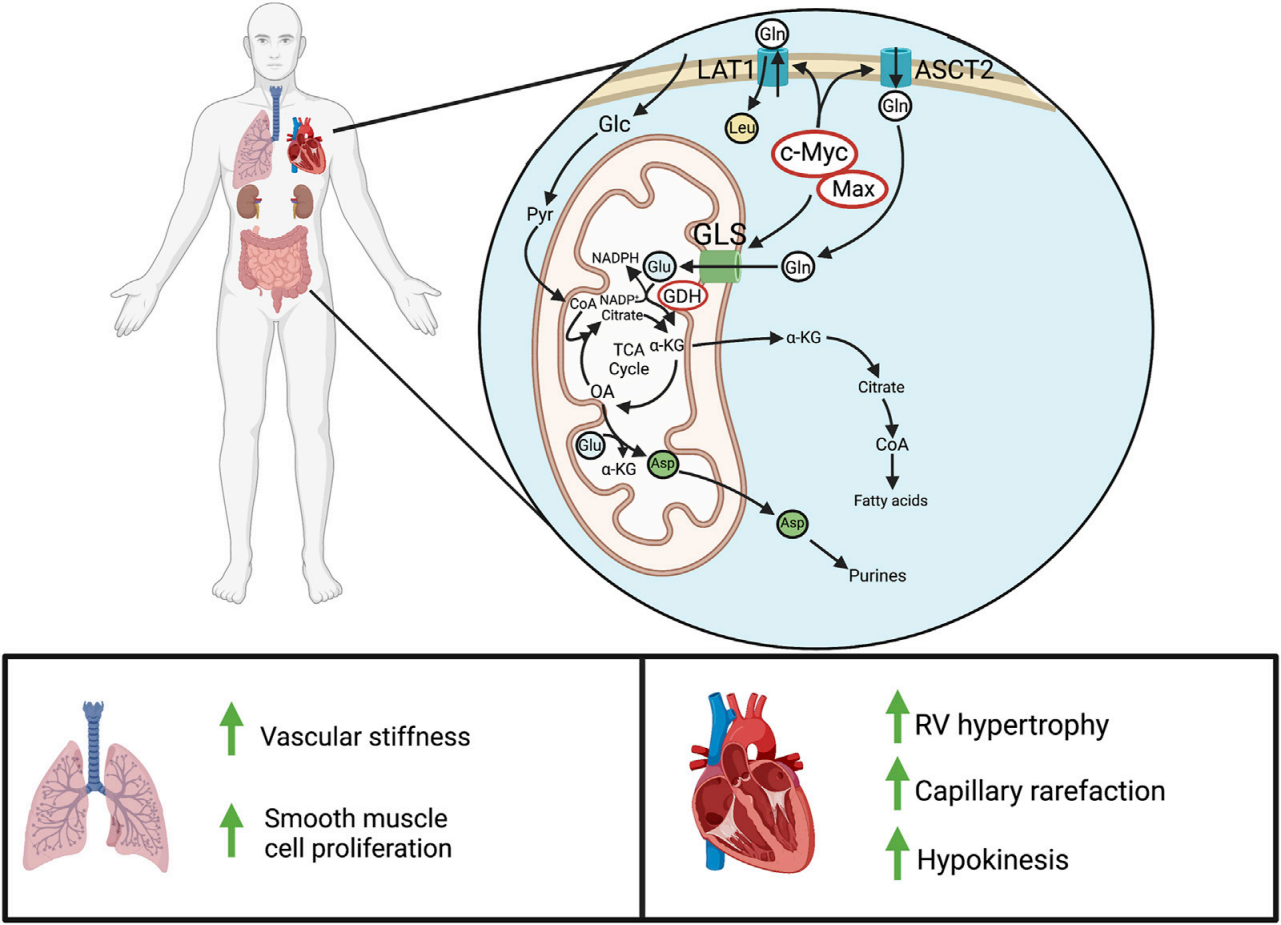
Glutaminolysis in PAH. Above: In normal adult cells, glutaminolysis predominantly occurs in actively proliferating cells, like those of the gastrointestinal tract and kidneys. In pathologic states of hyperproliferation, as occurs in cancer cells, there is also marked upregulation of glutaminolysis. Cellular glutamine entry is enhanced through c-Myc/Max-mediated transcription of the glutamine transporter, LAT1 (SLC7A5), and the ASCT2 (SLC1A5). GLS then produces glutamate for entry into the Krebs cycle, leading to α-ketoglutarate synthesis and downstream production of fatty acids, purines, and other metabolites. Below: In PAH, increased glutaminolysis in the pulmonary vasculature increases vascular stiffness and obstruction. In the normal RV, there is little or no basal glutaminolysis; however in PAH, cMyc and Max dramatically upregulate glutaminolysis. This contributes to RV hypertrophy and reduces RV function. Abbreviations: ASCT2—Alanine-serine-cysteine transporter 2; GLS—Glutaminase; GDH—Glutamate dehydrogenase; LAT1—L-type amino acid transporter 1; RV—Right ventricle.

Vascular stiffness secondary to endothelial dysfunction and increased collagen deposition in the extracellular matrix (ECM) is also associated with increased glutaminolysis in both PASMC and PAEC (Figure 4). This occurs *via* mechanosignaling through yes-associated protein 1 and transcriptional coactivator with PDZ-binding motif, which enhances glutaminase (GLS1) activity (Bertero et al., 2016). GLS1 is a mitochondrial matrix protein and serves an anapleurotic role, converting glutamine to glutamate before glutamate’s deamination by glutamate dehydrogenase into α-ketoglutarate and the antioxidant, NADPH. α-ketoglutarate may be converted into oxaloacetate, using a truncated form of the Krebs cycle, into purines for DNA synthesis, or citrate as a precursor for acetyl-CoA and fatty acids (Figure 4). Thus, glutaminolysis’ protean actions can feed the pseudo neoplastic phenotype of pulmonary vascular cells in PAH. Enhanced glutaminolysis thus contributes to the apoptosis resistance and increased cell cycle progression caused by Warburg metabolism and dysfunctional calcium transport in PAH. In the heart, the increased glutaminolysis appears to contribute to RV hypokinesis, perhaps through suppression of glucose oxidation, and to RVH.

As with other acquired mitochondrial abnormalities, increased glutaminolysis is evident in multiple cardiovascular cell types in PAH. PAH PAfib has a demonstrated increase in glutamine consumption (Bertero et al., 2016). Enhanced glutaminolysis has also recently been found to exacerbate ECM stiffening *via* α-ketoglutarate-mediated activation of mTOR and hydroxylation of PAfib-derived collagen proline residues, promoting collagen stability (Ge et al., 2018). In PAEC with a high-stiffness ECM, defined as a Young’s elastic modulus of ≥50 kPa, inhibition of GLS1 with either DON or *N,N’*-[Thiobis (2,1-ethanediyl-1,3,4,-thiadiazole-5,2,-diyl)]bisbenzeneacetamide (BPTES) reduced glutaminolysis, glycolysis, and cellular proliferation (Bertero et al., 2016). Inhaled telaglenastat, also an inhibitor of GLS1, has been shown to improve RV function, vascular remodeling, and hemodynamics in MCT-PAH when administered with verteporfin (Acharya et al., 2021).

Evidence also suggests that the progression of endothelial dysfunction in IPAH is intimately connected to increased glutaminolytic activity, as patients with *BMPR2* mutations have greatly reduced circulating glutamine levels (Egnatchik et al., 2017). Furthermore, PAEC with *BMPR2* mutations are intolerant of glutamine-depleted media, a phenomenon proposed to result from metabolic remodeling that shunts this amino acid into the Krebs cycle at higher rates to maintain energy reserves. Mitochondrial oxidant damage, mediated by mitochondrial ROS, has also been observed in these cells, which leads to isoketal accumulation and the inactivation of sirtuin-3, a mitochondrial deacetylase, collectively contributing to HIF-1α stabilization (Egnatchik et al., 2017). Glutamate receptor signaling is also implicated in disease progression in hyperproliferative diseases. For example, upregulation of the N-methyl-D-aspartate receptor (NMDAR) promotes cell proliferation in cancer (Li and Hanahan, 2013). Likewise, in both clinical and experimental PAH NMDARs are upregulated in PASMC (Dumas et al., 2018). PAH PASMC exhibit increased Ca^2+^-dependent glutamate release owing to downregulated Kv channels and activation of endothelin type A receptors (Dumas et al., 2018). Stimulation with PDGF, a mitogen that is upregulated in PAH, also leads to serine-896 GluN1 phosphorylation and enhanced cell proliferation in the chronic hypoxia model of PAH, and this is attenuated by the selective NMDAR blockers MK-801 and memantine (Dumas et al., 2018).

Collectively, these data indicate the existence of a largely unexplored and panvascular role of glutamine metabolism in PAH, contributing to vascular stiffening, maladaptive RVH, and endothelial dysfunction. Thus, the glutaminolysis pathway may offer important therapeutic targets and merits additional research.

### 2.5 Fatty acid oxidation

**FIGURE 5:**
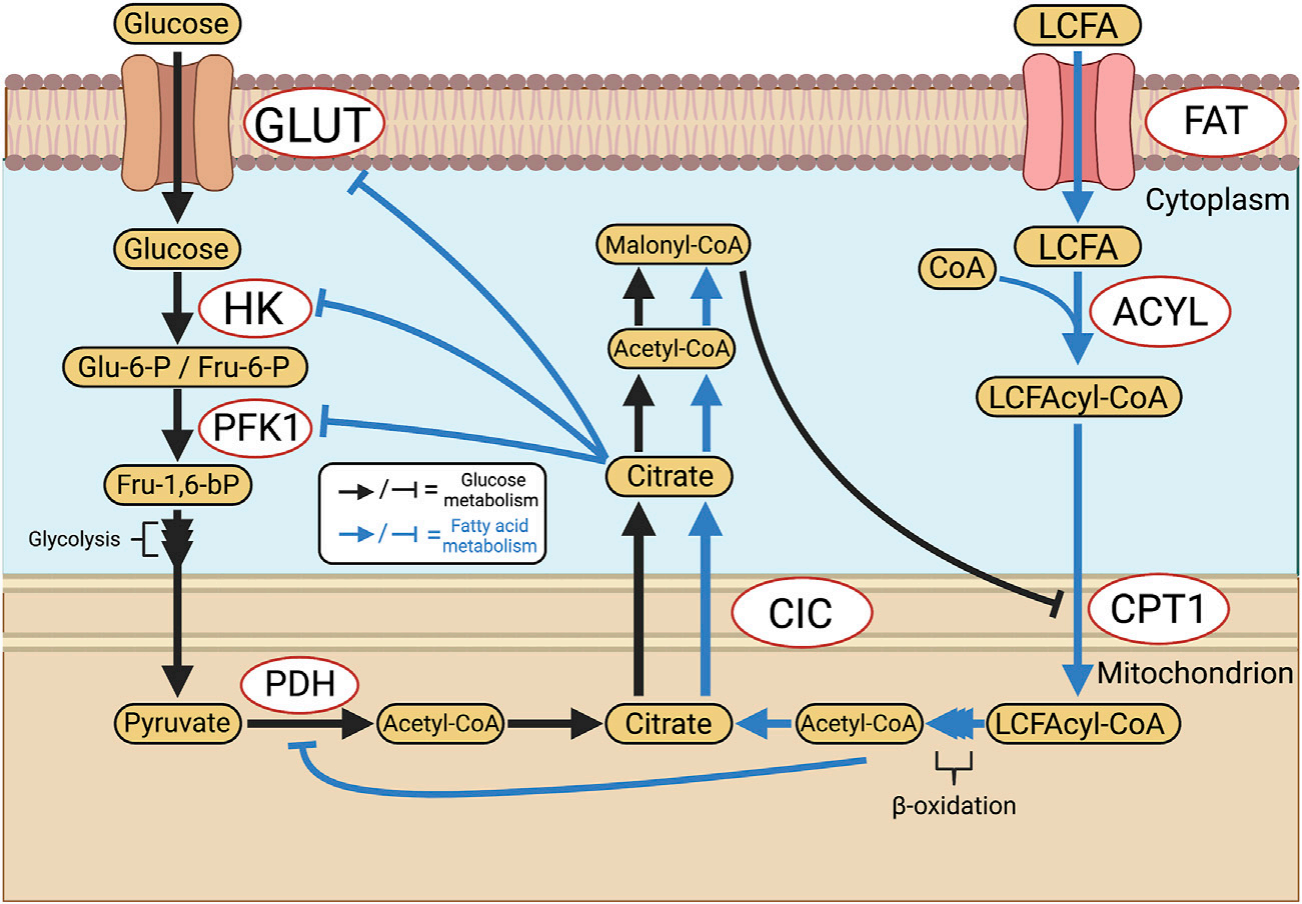
Reciprocal inhibition of glucose and fatty acid oxidation: the Randle Cycle. The Randle cycle refers to the phenomenon that products of GO inhibit FAO and *vice versa*. FAO is inhibited by GO at the level of CPT1 and the import of long-chain fatty acids into mitochondria, thereby increasing levels of free fatty acids. In the cytosol GO is inhibited by citrate, which reduces cytoplasmic glycolysis, whilst in the mitochondria PDH is inhibited by FAO-derived, mitochondrial, acetyl-CoA. Abbreviations: ACYL—Long chain fatty acyl-CoA ligase; CIC—Citrate carrier; CPT1—Carnitine palmitoyltransferase 1; FAT—Fatty acid translocase; GLUT—Glucose transporter; HK—Hexokinase; LCFA—Long-chain fatty acid; PDH—Pyruvate dehydrogenase; PFK1—Phosphofructokinase 1; TTP—Tricarboxylate transport protein.

FAO is another key function of mitochondrial metabolism that primarily plays a role in energy-intensive tissues such as the brain and heart. In the adult heart, FAO accounts for ∼60–90% of all ATP production, the remainder resulting from coupled glycolysis and GO (Neely and Morgan, 1974). The balance between GO and FAO is regulated, such that an increase in one form of oxidative metabolism reduces the other. This reciprocal relationship is referred to as the Randle cycle, named after its discoverer Phillip Randle (Randle et al., 1963). This dynamic leads to therapeutic opportunities in PAH, as FAO is less energetically efficient, producing approximately 4.6 moles of ATP per mole of oxygen compared to 5.2 *via* GO (Fillmore and Lopaschuk, 2013). Thus, glucose metabolism may be preferable to FAO in conditions of ischemia or hypoxia, even though less ATP is produced per mole of substrate. However, it is crucial to avoid uncoupled glycolysis since the by-product of this pathway, lactate, leads to intracellular acidosis, which impairs numerous cellular functions such as contraction in cardiomyocytes (Fillmore et al., 2018). The basis for the reciprocal relationship between GO and FAO relates to the production of acetyl-CoA from pyruvate *via* PDH (Figure 5). If acetyl-CoA is produced by FAO, acetyl-CoA and NADH accumulation inhibits PDH. In addition, the associated citrate production in the Krebs cycle during FAO inhibits phosphofructokinase-1 (PFK1) and hexokinase (HK). In aggregate, these FAO-induced changes inhibit GO. Conversely, GO-sourced acetyl-CoA is later converted into citrate by the Krebs cycle and exported from the mitochondrion by the tricarboxylate transport protein (TTP) (Infantino et al., 2011). Cytosolic acetyl-CoA is then formed from citrate *via* ATP-citrate lyase and is later converted into malonyl-CoA, which inhibits mitochondrial fatty acid import at the level of carnitine palmitoyltransferase (CPT1), an enzyme that normally supports the esterification and accumulation of long-chain fatty acids and triglycerides.

The Randle cycle, therefore, offers a therapeutic avenue to modulate GO versus FAO, and in the case of the hypertrophied, ischemic RV in PAH, one can partially inhibit FAO to shift metabolism in favor of GO. This can be done using drugs that are inhibitors of FAO which are in clinical use and could be repurposed from their use in patients with heart failure (trimetazidine) or angina (ranolazine). Previous studies identified Warburg metabolism in the RV in both preclinical and clinical forms of PAH and showed this was associated with ischemia due both to microvascular rarefaction and reduced epicardial perfusion pressure (Sutendra and Michelakis, 2014; Lahm et al., 2018; Tian et al., 2020a). Both pulmonary artery-banded and fawn-hooded rat models demonstrate increased FAO, which is targetable through FAO inhibitors such as ranolazine and trimetazidine, leading to improved cardiac output and regression of RVH in experimental (Fang et al., 2012) and clinical PAH (Khan et al., 2015). The use of partial inhibitors of FAO has potential translational relevance as both ranolazine and trimetazidine are approved for clinical use in patients with coronary artery disease and congestive heart failure, respectively. It has been suggested that these partial FAO inhibitors may inhibit sodium channels in cardiomyocyte (Belardinelli et al., 2006). However, our lab and others have shown that these drugs do inhibit FAO, theoretically at the level of 3-ketoacyl-CoA-thiolase (Kantor et al., 2000), without altering cardiomyocyte electrophysiology (Kantor et al., 2000; Fang et al., 2012).

While the adult right ventricle predominantly relies on FAO (Lopaschuk and Ussher, 2016), the Warburg shift observed throughout the cardiopulmonary unit in PAH reverts its metabolic profile to a phenotype more reflective of the fetal RV, where glucose is the primary substrate for metabolism. Indeed, fatty acid metabolism is radically altered in the RV of PAH patients. Circulating free fatty acids and acylcarnitines, which are necessary for fatty acid membrane transport, are increased approximately 2-fold and 1.3-fold in PAH, respectively (Brittain et al., 2016). Fatty acid accumulation in cardiomyocytes is connected with cardiac steatosis, insulin resistance and lipotoxicity, predicting poor prognosis (Heresi et al., 2010; Pugh et al., 2011). Furthermore, in BMPR2-knockout mouse models of PAH, increased triglyceride and ceramide concentrations are observed in RV tissue, which is relevant because ceramide can induce heart failure (Hemnes et al., 2014). Reduction of *de novo* fatty acid production by blocking fatty acid synthase (FAS) using siRNA or C75, a FAS inhibitor, reverses PAH progression in the RV of animal models*,* demonstrating that fatty acid synthesis constitutes a potential therapeutic target in PAH (Singh et al., 2016). While the precise contributions of altered FAO in PAH pathogenesis likely vary amongst PAH subtypes and between animal models, abnormal FAO in PAH reduces RV function and contributes to increased cell proliferation. Interestingly we find that FAO is upregulated in adaptive RVH, as induced in rats by pulmonary artery banding (Fang et al., 2012), where it is downregulated in PAH, as occurs in fawn hooded rats, which spontaneously develop PAH (Michelakis et al., 2002a). The Randle cycle is a promising therapeutic target in PAH.

## 3 The emerging role of mitochondrial dynamics in PAH

### 3.1 The processes of mitochondrial dynamics

**FIGURE 6:**
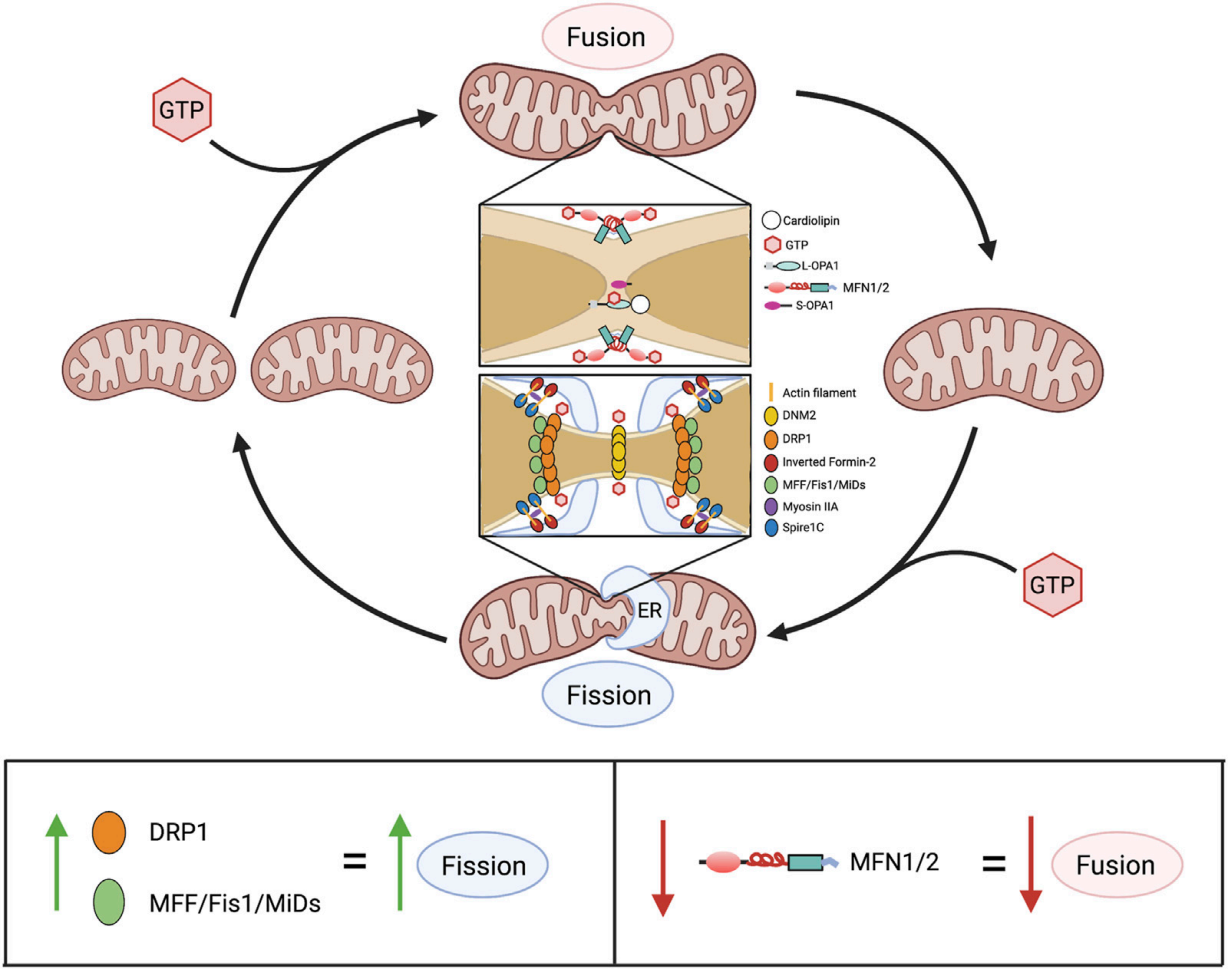
Overview of mitochondrial dynamics. Above: Mitochondrial fusion and fission are GTP-dependent mechanisms that cause mitochondria to join together and divide, respectively. In fusion, association between two adjacent mitochondria is initially made *via* the cytoplasm-facing, C-terminal heptad repeat domains of MFN1 and/or MFN2. This produces a coiled-coil structure, with GTP hydrolysis by an N-terminal GTPase domain drawing adjacent outer mitochondrial membranes closer together, enabling fusion. Subsequently, GTP hydrolysis by L-OPA1, in L-OPA1-cardiolipin associations, enables fusion of the inner membranes, finalizing the union between mitochondria. In fission, serine-616-phosphorylated DRP1 (the activated form of DRP1) moves from the cytosol to the outer mitochondrial membrane where it binds one or more of its adaptor proteins (MFF, Fis1, MiD49 and MiD51), which are anchored on the outer mitochondrial membrane. This formation of fission rings occurs at sites with increased mitochondrial DNA (not shown) that are demarcated by association with the ER. Formation of DRP1 oligomers and their interaction with ER and actin filament structures, including spire1c and inverted formin-2, contribute to the fission machinery. Multimers of DRP1 and the other fission-mediating proteins encircle the mitochondria. The fission apparatus constricts following DRP1 GTP hydrolysis. DNM2, which creates a fission apparatus of even smaller diameter than DRP1, is postulated to complete the fission process, although this remains controversial. Below: Alterations in expression of mitochondrial dynamics proteins and consequences for the fusion-fission balance. Abbreviations: DNM2—Dynamin 2; DRP1—Dynamin-related protein 1; ER—Endoplasmic reticulum; Fis1—Fission protein 1; GTP—Guanosine triphosphate; MFF—Mitochondrial fission factor; MFN1/2—Mitofusin 1/2; MiDs—Mitochondrial dynamics proteins of 49 and 51 kDa; L/S-OPA1—Long/short optic atrophy one.

In addition to regulating calcium concentrations and metabolism, mitochondria have non-canonical functions which in aggregate are referred to as mitochondrial dynamics. This term recognizes that mitochondria exist in networks, wherein they regularly join and divide by processes called fusion and fission, respectively (Figure 6). These activities serve context-dependent functions (ranging from quality control and bioenergetics to coordination of mitochondrial and nuclear division—a process called mitotic fission), as previously reviewed (Archer, 2013). Mitochondria also move within the cell along microfilaments, a dynamic process regulated by Miro and Milton (Barnhart, 2016). Both fusion and fission are executed *via* large GTPases. Mitofusins 1 (MFN1) and 2 (MFN2) are responsible for the fusion of the OMM (Chen et al., 2003), while optic atrophy 1 (OPA1) controls the fusion of the IMM (Chen et al., 2003; Cipolat et al., 2004). DRP1 is the principal mediator of mitochondrial fission, and in humans, is functionally regulated by a vast array of post-translational modifications including phosphorylation, SUMOylation, and ubiquitination (Frank et al., 2001).

#### 3.1.1 Mitochondrial fusion

Mitochondrial fusion was first discovered in *Drosophila melanogaster*, wherein fuzzy onions (FZO), a mitofusin homolog, aids in the process of spermatogenesis *via* fusion and remodeling of spermatid mitochondria (Hales and Fuller, 1997). Fusion is thought to allow the mixing of OXPHOS subunit-encoding mitochondrial DNA (mtDNA), allowing maximal participation of a cell’s mitochondria in ATP production during stress or other periods of high energy demand (Westermann, 2012). The discovery of FZO was seminal in the mitofusin field, with conserved homologs later being found across eukaryotes, including all mammals (Rapaport et al., 1998; Santel and Fuller, 2001). When first discovered, MFN2 was also referred to as *hyperplasia suppressor gene* because of its antiproliferative effect in SMC (Chen et al., 2004).

**FIGURE 7:**
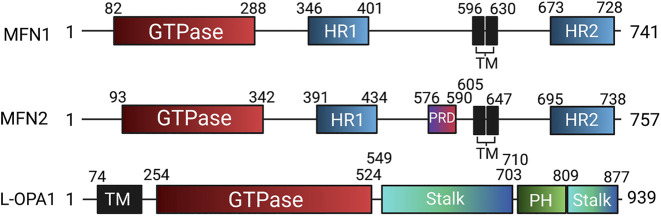
Domain organization of human mitochondrial fusion mediators. Human mitofusin 1 (MFN1), mitofusin 2 (MFN2), and long optic atrophy 1 (L-OPA1) with annotated domain boundaries. Abbreviations: GED—GTPase effector domain; HR - Heptad repeat; HRN - N-terminal truncated heptad repeat, PH - Pleckstrin homology; PRD—Proline rich domain; TM - Transmembrane.

In humans, the FZO homologs MFN1 and MFN2 are structurally similar to one another, though MFN2 bears a proline-rich domain which is believed to assist in protein-protein interaction (Figure 7). Otherwise, the mitofusins consist of an N-terminal catalytic GTPase domain and two heptad repeats separated by two transmembrane domains, with MFN1 being 741 residues long and MFN2 757. The largely accepted topology of MFN expression places them on the OMM as either homo- or heterodimers which initiate mitochondrial fusion through their cytosol-facing C-terminal heptad repeat domains. *Trans* associations between MFN1 and MFN2 heptad repeat domains have greater fusogenic efficacy than homodimeric associations and produce a more stable, antiparallel coiled-coil arrangement (Koshiba et al., 2004; Hoppins et al., 2011). This coiled-coil association draws the outer membranes of distinct mitochondria into closer proximity prior to lipid bilayer mixing. However, recent work by Mattie *et al.* indicates that in humans, the C-terminal heptad repeat domain 2 resides in the intermembranous space, giving MFNs an “N_out_-C_in_” topology akin to TOM20 and Mim1/Mim2, where the N-terminus faces the cytosol, and the C-terminus exists in the intermembrane space. Further, conserved cysteine-684 and -700 residues within this region act as sensors for oxidative stress and enable dimerization that precedes fusion (Mattie et al., 2018). These findings suggest that the MFN HR1 and HR2 domains may be compartmentally separated in opposition to the accepted topology, underscoring the imperfect understanding of the fusion mechanism. GTPase assays also indicate that MFN1 has approximately 8-fold the GTP turnover rate of MFN2, reflecting distinct and incompletely defined roles of these enzymes in the fusion process (Ishihara et al., 2004). For instance, following outer membrane fusion, OPA1, which is expressed on the IMM, is dependent on MFN1 for its activity (Cipolat et al., 2004). After a GTP-dependent conformational change in MFN1 and MFN2, which connects the outer membranes, OPA1 may complete fusion through several possible mechanisms. OPA1 exists in long and short variants, respectively known as L- and S-OPA1 (Song et al., 2007). Under basal conditions, the mitochondrial inner membrane i-AAA protease, YME1L, cleaves L-OPA1 at its transmembrane domain to produce S-OPA1, leading to steady-state populations of both variants (Ehses et al., 2009; Head et al., 2009). During stress, the IMM protease OMA1 enhances L-OPA1 processing (Anand et al., 2014). As a consequence, the fusion-inactive S-OPA1 becomes more prevalent in supporting fragmentation of the mitochondrial network (Anand et al., 2014). As with MFN1 and MFN2, the established mechanism for IMM fusion involves interactions between embedded L-OPA1 proteins on neighboring mitochondria. However, a recent study has built upon prior observations that L-OPA1 knockout mitochondria were still able to undergo fusion. This appears to be enabled through the heterotypic association between L-OPA1-competent mitochondria and cardiolipin, a lipid that is highly enriched in the IMM, within adjacent L-OPA1-deficient mitochondria (Ban et al., 2018).

The variety of fusion mechanisms exhibited by mitochondria is reflected in the diversity of cell contexts in which the process occurs. Mitochondrial fusion is a key quality control process whereby damage to the organellar membrane or genome of a given mitochondrion can be dissipated by complementation with a fusion partner (Nakada et al., 2001; Yang et al., 2015). Many clinical phenotypes related to mitochondrial dysfunction may present when a threshold of damage is exceeded. A classic example of fusion’s quality control function was demonstrated by Nakada *et al*. They performed electrofusion on mouse zygotes using somatic cells bearing mitochondrial DNA with a 4696-base pair deletion (∆mtDNA4696). This was anticipated to yield a loss of OXPHOS capacity at the level of cytochrome c oxidase (COX). Although there was 66% prevalence of ∆mtDNA4696-bearing mitochondria in skeletal muscle and kidneys, these tissues still maintained normal COX activity (Nakada et al., 2001). Respiration deficiency-related mitochondrial dysfunction, resulting in renal failure and elongated cardiac PQ intervals, was only manifested in mice with >85% of cells bearing ∆mtDNA4696, in which case complete COX deficiency was observed (Nakada et al., 2001). These data showed that host COX-positive mitochondria can compensate for COX-negative organelles by a mixing of matrix contents, thus minimizing mitochondrial damage and loss of function, provided the prevalence of mutant mtDNA does not exceed a threshold.

#### 3.1.2 Mitochondrial fission

DRP1 is the chief fission mediator and executes its fissile activity to achieve a variety of different downstream effects, which include regulation of cell death, the production of ROS, induction of apoptosis, and the coordination of mitochondrial and nuclear division (mitotic fission). DRP1 is a member of the dynamin-like protein family. The dynamins, dynamin 1 (DNM1), 2 (DNM2), and 3 (DNM3), play varied roles in regulating the structure of biological membranes, ranging from mediating clathrin-associated endocytosis to actin cytoskeleton associations that support cell adhesion and motility, reviewed in (Ferguson and De Camilli, 2012). Five DRP homologs have been identified in plants, including DRP2-4, DRP5A, and DRP5B. These DRPs mediate the division/fission of plant organelles, including mitochondria, peroxisomes and chloroplasts. DRP3A/3B are the first reported homologous proteins known to function in both peroxisomal and mitochondrial division in *Arabidopsis* (Arimura et al., 2004; Logan et al., 2004; Mano et al., 2004; Fujimoto et al., 2009; Zhang and Hu, 2009). DRP5B, a protein distantly related to DRP3, contributes to both chloroplast division and peroxisome division in *Arabidopsis* (Zhang and Hu, 2010). Unlike DRP3A and DRP3B, whose orthologs exist across plant, fungal, and animal kingdoms, DRP5B is a plant- and algae-specific DRP (Zhang and Hu, 2010). In metazoans, DRP1 exists in 1 of nine isoforms, and while the exon sequence of each has been described, current health science literature rarely accounts for isoform specificity (Rosdah et al., 2020). DRP1 includes an N-terminal bundle signaling element (BSE) and GTPase domain, the latter of which may be phosphorylated by glycogen synthase kinase 3*β* at serines 40 and 44 to enhance its catalytic activity (Yan et al., 2015). Downstream of the GTPase domain are a middle domain and a C-terminal GTPase effector domain (GED), which are key to the assembly of higher-order DRP1 structures that mediate fission (Zhu et al., 2004; Ramachandran et al., 2007). Intersecting the middle and GED is a B domain, which contains multiple SUMOylation sites that can inhibit DRP1 activity (Figueroa-Romero et al., 2009). Critically, each DRP1 isoform possesses phosphorylation sites at serine 616 and serine 637 in the GED, which are targets for numerous kinases and phosphatases that regulate DRP1 activity (Yapa et al., 2021).

In its inactive state, DRP1 is a cytoplasmic protein that exists in monomeric, dimeric, and tetrameric forms. There remains debate about the role of serine 637 phosphorylation status in DRP1 activation (Yu et al., 2019a), although phosphorylation at this site is thought to inhibit fission. In contrast, DRP1 phosphorylation at serine 616, which is executed by many kinases, including cyclin-dependent kinase 1 (CDK1), is a well-established mechanism for DRP1 activation, leading to its translocation to the OMM (Taguchi et al., 2007; Michalska et al., 2018). The precise localization of DRP1 on the OMM is dictated by contact sites between the endoplasmic reticulum tubules and the OMM (Friedman et al., 2011), with co-localization of inverted formin-2 (Korobova et al., 2013) and Spire1c (Manor et al., 2015) serving to polymerize actin filaments which assist in DRP1-mediated mitochondrial constriction. While DRP1 does not have a lipid-binding domain, it is able to coordinate with the mitochondrial cardiolipin in the early stages of fission (Bustillo-Zabalbeitia et al., 2014). DRP1 also binds to several receptor proteins on the OMM which serve to localize and coordinate its macromolecular assembly into a fission ring, including mitochondrial fission factor (MFF), fission protein 1 (FIS1), and mitochondrial dynamics proteins of 49 kDa (MiD49) and 51 kDa (MiD51). While the precise roles of each DRP1 binding partner are not fully understood, MFF is thought to contribute substantially to the association between activated DRP1 and the OMM (Otera et al., 2010), and FIS1 plays roles both in fission mediation and fusion inhibition (Yu et al., 2019b). By interacting with its adaptor proteins and the actin cytoskeleton, DRP1 forms higher-order ring-like structures at the OMM and consumes GTP to induce a contractile force, leading to mitochondrial fission. There is debate over whether DNM2, another GTPase, exists as a secondary fission mediator, performing the final stage of scission following DRP1 activity (Lee et al., 2016; Fonseca et al., 2019). *In vitro* experiments in various human cancer cell lines have shown that DNM2 knockdown produces both enhanced fusion of the mitochondrial network and constricted-yet-undivided regions on mitochondria (Lee et al., 2016), which suggests a role for DNM2 in fission. However, arguing against a universal role for DNM2 is the observation that DNM1-3 triple knockdown in mouse fibroblasts did not alter fission of either mitochondria or peroxisomes (Fonseca et al., 2019). The role of DNM2 in mitochondrial fission remains controversial and is an active area of investigation in our laboratory.

Recent insight into the interaction between DRP1 and its adaptors MiD49/MiD51 suggests that both DRP1 and MiD49 can interact with up to four binding partners (Kalia et al., 2018). Cryogenic electron microscopy structures reveal a series of sites within the GTPase and stalk domains of DRP1 that can bind to MiD49/51 (Kalia et al., 2018). However, only two of these sites are available prior to GTP association, with the remaining two being revealed through conformational changes in the N-terminal BSE and GTPase domain (Kalia et al., 2018). MiD49 can associate with multiple molecules of GTP-bound DRP1 through numerous salt bridge interactions, requiring a conserved dynamin recruitment region (Kalia et al., 2018). Indeed, the fission mechanism and the roles of each protein involved are only beginning to be understood and present a promising area for future research.

Fission regulates several roles in cellular function, including mitophagy, a mechanism of mitochondrial quality control. Following DRP1-mediated fission, PTEN-induced kinase 1 (PINK1) and E3 ubiquitin-protein ligase known as Parkin are sequentially directed to the OMM. PINK1 is typically degraded by the mitochondrial processing peptidase (MPP) and presenilin-associated rhomboid-like protease (PARL) (Greene et al., 2012). However, during mitophagy, MPP and PARL are inhibited prior to PINK1’s OMM association, allowing it to recruit Parkin and ubiquitinate voltage-dependent anion channels, as well as MFN1 and MFN2 (Greene et al., 2012). This ubiquitination supports autophagosome formation and subsequent mitochondrial degradation by lysosomal hydrolases. The significance of the PINK1-Parkin signaling axis is made clear by its hyperactivation *via* PARL inhibition, which exists as a risk factor for Parkinson’s disease (Meissner et al., 2015). In Parkinson’s disease, increased DRP1-mediated fragmentation of mitochondria in dopaminergic neurons leads to cell death and neurodegeneration (Filichia et al., 2016). In the heart, DRP1 activity during ischemia/reperfusion contributes to cardiomyocyte apoptosis (Ong et al., 2010), though in health, a basal level of fission may be cardioprotective (Ikeda et al., 2015). In cardiac-specific DRP1 knockout mice, the ability of mitochondria to respond to glucose deprivation and undergo quality control through mitophagy is impaired, ultimately leading to reduced ATP production, enhanced apoptosis, and myocardial fibrosis (Ikeda et al., 2015).

Alongside its pivotal role in cellular quality control, mitochondrial fission is necessary for organellar biogenesis. The mechanistic distinction between these two processes is only beginning to be unraveled, but recent work has shown that mitophagy and biogenesis are separated by the physical localization of a fission event within the mitochondrion. It appears that mitophagy follows peripheral fission, denoted by a division of a mitochondrion at its periphery, while mitochondrial biogenesis is initiated through central fission events, dividing a given mitochondrion into daughter organelles of roughly equal size (Kleele et al., 2021). Kleele *et al*. found that the central division of mitochondria (relevant to mitotic fission) is regulated by DRP1 binding to MFF and is localized to points of ER contact; whereas peripheral division (relevant to mitophagy) is regulated by DRP1 binding to FIS1 and is preceded by lysosomal contact. Furthermore, endoplasmic reticulum, actin, and MFF association are not observed in peripheral fission, the form of fission common in mitophagy, which occurs downstream of stressors such as increased ROS and a loss of the OXPHOS-driving proton motive force (Kleele et al., 2021). Interestingly, peroxisome proliferator-activated receptor-γ coactivator-1α (PGC-1α) is a major regulator of mitochondrial biogenesis, while also controlling the transcription of MFN2 and the nuclear respiratory factors NRF1 and NRF2, thus giving PGC-1α roles in both fission and fusion processes (Gureev et al., 2019). Upstream of mitochondrial fission, stimuli such as oxidative stress activate PGC-1α, in turn, upregulate NRF1 and NRF2 and promote their translocation to the nucleus where they act as transcription factors for genes that assist in mtDNA transcription, such as mitochondrial transcription factor A, otherwise known as TFAM, and B1. Increases in mtDNA have been found to predict sites of endoplasmic reticulum-OMM association, suggesting a connection between DRP1-mediated fission and mtDNA replication (Lewis et al., 2016). Kleele *et al*. found that this role for mtDNA was true for mid-zone but not peripheral fission.

### 3.2 Mitochondrial dynamics and the cell cycle

The dynamism of the mitochondrial network is intimately connected to both a cell’s health and proliferative state throughout the cell cycle. Mitochondrial fission is a key event in mitosis and ensures their equitable distribution between daughter cells, while hyperfusion of the network supports increased ATP production and allows entry of cells into S phase (Mitra et al., 2009; Westermann, 2012). However, if MFN2 is overexpressed, this too causes inhibition of cell proliferation and cell cycle arrest; a reminder of the important and changing balance between fission and fusion at the varying stages of cell division (Rehman et al., 2012; Dasgupta et al., 2021).

Throughout the cell cycle, the mitochondrial network is altered, reflecting dynamic changes in fusion and fission, as well as the activity of the actin cytoskeleton, microtubules, and their associated motor proteins, such as kinesins and dyneins. Fission and fusion are also regulated by mitochondrial lipids, like cardiolipin and phosphatidic acid (Frohman, 2015). During the state of quiescence that constitutes the G_0_ phase, mitochondria exist in both fragmented and fused morphologies (Antico Arciuch et al., 2012). Cell cycle entry into the G_1_ phase, by means of growth factor receptor activation or other extracellular stimuli, prompts a rebalancing of the mitochondrial network into a more highly fused structure, enhancing oxygen uptake and ATP production (Mitra et al., 2009). Synthesis of nuclear and mitochondrial DNA is enhanced during this time, in parallel with the transcription of CDKs and the accumulation of cyclin E, which is necessary for a cell to pass through the G_1_-S checkpoint (Mitra et al., 2009). Once in the G_2_ phase, however, increasing levels of mitochondrial fragmentation are observed, reflecting that the cell is now fully committed to undergoing division. Indeed, the CDK1-cyclin B1 complex, which activates DRP1 through phosphorylation at serine-616, permits increased fission and progression through the G_2_/M checkpoint. This complex additionally contains the small Ras-like GTPase RALA and its effector RALBP1, which is phosphorylated by aurora A kinase, allowing for RALA-RALBP1 relocation to the OMM, enhancing DRP1 phosphorylation and mitochondrial recruitment ∼18-fold (Kashatus et al., 2011). Interestingly, MFN2 overexpression in mouse embryonic fibroblasts reduces levels of total DRP1, as well as MFN1 and OPA1 while inducing G_2_/M cell cycle arrest, suggesting additional, undetermined signaling relationships amongst mitochondrial dynamics proteins (Chen et al., 2014).

Mitochondria have a multifaceted relationship with cytoskeletal components such as actin filaments and microtubules. While actin filaments are necessary for the preconstriction step in mitochondrial fission—being recruited by inverted formin 2 on the ER (Korobova et al., 2013) and Spire1c on the mitochondria (Manor et al., 2015)—as well as allowing mitochondrial motility in yeast, microtubules are the key structure that allow mitochondria to move in three-dimensional space in mammalian cells. Critical to mammalian mitochondrial motility are the OMM-anchored proteins Miro1 and Miro2, a subclass of Ras monomeric GTPases. Miro proteins are bound to the OMM by a C-terminal transmembrane domain, and feature both N- and C-terminal GTPase domains, a structural property that is unique amongst all human GTPases. While the precise roles of these GTPase domains in mitochondrial trafficking are imperfectly understood, Miros have robustly been shown to recruit trafficking kinesin-binding protein 1 (TRAK1) and 2 (TRAK2), mammalian homologs of Milton in *D. melanogaster*, *via* the N-terminal domain (Glater et al., 2006). These adaptors are capable of binding both kinesin-1 and dynein, though TRAK2 preferentially interacts with dynein, enabling anterograde and retrograde movement of mitochondria, respectively. During cytokinesis, Miro-TRAK1/2-motor protein complexes recruit kinetochore protein F, which facilitates nuclear envelope breakdown, and enables anterograde movement of mitochondria towards the cell’s cleavage furrow, potentially supplying ATP for contraction of the actomyosin ring (Lawrence and Mandato, 2013). Alternatively, mitochondria may associate with dynein and its activator dynactin for retrograde trafficking, allowing organellar degradation or their homogenous distribution throughout a cell, though to date, these functions have largely been explored in neurons (Mandal and Drerup, 2019; Mandal et al., 2021). Other inter-organellar associations, particularly the mitochondria-associated membranes (MAMs) existing between mitochondria and the endoplasmic reticulum, are believed to be uniquely modulated by MFN2 (de Brito and Scorrano, 2008) and regulate calcium-mediated control over the cell cycle. The precise role of MFN2 in facilitating interaction between these subcellular structures is unknown, though there is debate about whether it serves as an enhancer (Schneeberger et al., 2013; Sugiura et al., 2013) or a repressor (Filadi et al., 2016; Leal et al., 2016) of tethering. While electron microscopy studies suggest that MFN2 reduces MAM formation (Cosson et al., 2012), recent *in vivo* work in mouse hippocampal neurons shows that MFN2 expression is critical for both close and longer-range contacts between these organelles (Han et al., 2021). Close-contact MAMs provide the majority of Ca^2+^ and phospholipid transfer between the ER and mitochondria, and the proteins which facilitate such transfer, like Grp75, VDAC1, and IP3R3, are reduced in expression following MFN2 knockout (Han et al., 2021).

Reflective of the cell cycle’s sensitivity to the balance of mitochondrial fusion and fission, mitochondrial dynamics are closely connected to cell viability. Depending on the cellular context, both fusion and fission have the potential to be pro-apoptotic or may act in support of cell division and survival. As previously discussed, mitochondrial fusion enables optimal ATP production and allows movement through the G_1_-S cell cycle checkpoint *via* enhanced cyclin E stability (Mitra et al., 2009). Fusion is also enhanced during starvation (Gomes et al., 2011) and may play a role in cell survival. During starvation, the mitochondrial network tubularizes *via* reduced phosphorylation of DRP1 at serine-616, concurrent with increased phosphorylation at serine-637 (Rambold et al., 2011). Such tubular mitochondria, which have enhanced ATP synthase activity (Gomes et al., 2011), are also spared from autophagosomal degradation and may donate lipids for vesicle formation (Rambold et al., 2011). This aids in the procurement of nutrients from other cellular sources as a pro-survival mechanism. However, a large body of literature also shows that fusion mediators like MFN2 are key in promoting cellular quiescence and apoptosis sensitivity (Guo et al., 2007; Shen et al., 2007; Rehman et al., 2012). This regulatory function, at least in the case of MFN2, has been proposed to occur *via* multiple mechanisms. These include mitochondria localization-independent association of MFN2’s N-terminal region (residues 1–264) with Raf1, which reduces phosphorylation of ERK, as well as localization-dependent C-terminal (residues 265–700) interaction with Ras (Chen et al., 2014). Both features result in cell cycle arrest at the G_2_/M phase. In lung cancer, MFN2 has also been demonstrated to inhibit Rictor, a subunit of the mTORC2 complex, which prevents AKT1 phosphorylation and subsequent cytoskeletal reorganization for cell division (Xu et al., 2017). Mitochondrial fission also bears a context-dependent relationship to cell survival. In healthy cells, fission is involved in a dynamic balance between being pro-apoptotic and pro-proliferative. This is due to the role of mitochondria as a critical gatekeeper of intrinsic cell death, with oligomerized Bax and Bak SUMOylating DRP1 to enhance associative stability at the OMM before cytochrome c release and caspase activation (Wasiak et al., 2007). Accordingly, knockout of DRP1 prevents apoptosis-related mitochondrial fragmentation even when Bax is overexpressed (Frank et al., 2001). The fission machinery therefore acts as a mediator of programmed cell death, which can be activated following stimuli such as oxidative stress, as in cardiac ischemia-reperfusion injury (Wu et al., 2020). However, in diseases of hyperproliferation, the dynamic balance of functions that fission performs is skewed towards mitotic fission, wherein mitochondrial division precedes nuclear division.

### 3.3 Mitochondrial dynamics in PAH

A growing body of literature has identified that DRP1-mediated mitochondrial fission is upregulated in both cells of the pulmonary vasculature (Marsboom et al., 2012a) and in the RV in PAH (Tian et al., 2018). Indeed, our laboratory has found upregulated expression of DRP1 in both PAH and cancer cells with a parallel decrease in the expression of MFN2 in both PAH PASMC (Marsboom et al., 2012a) and lung cancers (Rehman et al., 2012). Upregulation of DRP1 has been shown to play a role in obstructive hyperproliferation and RVH that underlies disease worsening (Abu-Hanna et al., 2018; Tian et al., 2018). In both experimental and human PAH, total DRP1 and activated serine 616-phosphorylated DRP1 levels are increased in PASMC. Inhibition of DRP1 activity, using Mdivi-1 or siDRP1, reduces rates of proliferation and induces cell cycle arrest (Marsboom et al., 2012a). Pharmacological enhancement of the inactive form of DRP1, pDRP6_S637_, by treprostinil, a prostacyclin analog, also increases mitochondrial fusion in PAH PASMC (Abu-Hanna et al., 2018).

While DRP1 is upregulated and activated in human and experimental PAH, MFN2 and its transcriptional coactivator PGC-1α are downregulated in IPAH, as well as in the MCT-PAH and Su/Hx-PAH models (Ryan et al., 2013). MFN2’s pivotal role in mitochondrial fusion underlies an inverse correlation between its expression and PAH disease severity. siMFN2 treatment reduces both MFN2 and PGC-1α expression, resulting in a concurrent increase in rates of PASMC proliferation. Conversely, adenovirus-mediated overexpression of MFN2 restores MFN2 expression in the lung while slowing PASMC proliferation and regressing PAH, evident as improved pulmonary vascular hemodynamics in female Su/Hx-PAH rats (Ryan et al., 2013). The downregulation of MFN2 in PAH as well as lung cancer, in part, appears to be tied to increased proteasomal degradation, which is triggered by PINK1-induced serine-442 MFN2 phosphorylation (Dasgupta et al., 2021). Fusion of the mitochondrial network through MFN2 restoration leads to G_0_/G_1_ cell cycle arrest in human PASMC, highlighting the critical yet incompletely understood mechanism of MFN2 in mitotic fission (Dasgupta et al., 2021).

Like DRP1 itself, the DRP1 adaptor proteins FIS1 (Marsboom et al., 2012a) and MiD49/51 (Chen et al., 2018) have each been shown to have increased expression in PAH. Indeed the exm1300952 single-nucleotide polymorphism in *SMCR7*, the gene encoding MiD49, is associated with pre- and post-capillary pulmonary hypertension (Assad et al., 2016). MiD proteins are upregulated in both animal and human PAH because of miR-34a-3p downregulation in PASMC (Chen et al., 2018). Plasma levels of miR-34-a-3p may also have merit as a diagnostic biomarker in PAH. On a therapeutic level, nebulization with siMiD49/51 or a miR-34a-3p mimic is effective in inhibiting mitochondrial fission and regresses MCT-PAH, regressing distal muscularization of small pulmonary arteries and improving hemodynamics, as measured by significant reduction in mPAP and total pulmonary resistance (Chen et al., 2018). MiDs appear to control cell proliferation by multiple mechanisms, including an incompletely understood effect on the phosphorylation of CDK4, the ability to enhance the expression of PDGF receptors α and β, and by allowing cell cycle progression by increasing ERK activity (Chen et al., 2018).

The normoxic activation of HIF-1α by cobalt chloride leads to pulmonary hypertension *in vivo* and in PASMC, and increases fission, fragmenting the mitochondrial network (Marsboom et al., 2012a). Treatment with Mdivi-1, a DRP1 GTPase inhibitor developed by the Nunnari lab (Cassidy-Stone et al., 2008), has proven effective in regressing PASMC proliferation in this model both at the *in vitro* and *in vivo* levels, leading to improvements in exercise capacity and PVR and inducing a more elongated mitochondrial network (Marsboom et al., 2012a). However, Mdivi-1 has imperfect specificity and can lead to downregulation of the mitochondrial fusion mediator L-OPA1, which may limit its potential for clinical use (Aishwarya et al., 2020). Drpitor1a, a small molecule, ellipticine, DRP1 GTPase inhibitor, developed in our lab, is specific for DRP1 and is 50-fold more potent than Mdivi-1. Drpitor1a has shown merit in improving several hallmark features of cancer (Wu et al., 2020). *In vitro* testing in PASMC revealed that Drpitor1a reduces proliferation rates. Likewise, in cancer cells and xenotransplant tumors, Drpitor1a slows cancer cell proliferation, induces apoptosis, and shrinks tumors without interfering with related GTPases such as DNM1, suggesting its high specificity as a Drp1 inhibitor (Wu et al., 2020). In preliminary studies in MCT-PAH, Drpitor1a also can regress PAH and protect the RV (Wu et al., 2021).

Mitochondrial dynamics are altered in PAH across multiple cell types. Recent evidence suggests that in PAEC, the dysregulation of DRP1-mediated fission impacts mitochondrial calcium stores and supports cell migration, potentially contributing to apoptosis resistance and the formation of a neointima (Shen et al., 2015). Derangements of DRP1 activity extend to the heart, with RV dysfunction and increased RV end-diastolic pressure (RVEDP) recently being linked to increased mitochondrial fission in MCT-PAH (Tian et al., 2017). Inhibiting DRP1 activity by Mdivi-1, P110 (an inhibitory peptide that blocks Fis1-DRP1 interaction), or Drpitor1a reduces the elevation of RVEDP that follows RV ischemia-reperfusion injury and reduces ROS production (Tian et al., 2017; Wu et al., 2020).

In healthy cardiomyocytes, mitochondrial networking differs from that seen in vascular tissue. Vascular cells typically demonstrate extensive fusion and filamentous organization, while cardiomyocytes have compartmentalized mitochondria that remain relatively static and spheroidal within/between myofibrils (Vendelin et al., 2005). Additionally, the fusion-fission cycle in cardiomyocytes is slow, reported to last approximately 2 weeks in adult mice, whereas that of human neurons has been shown to last approximately 20–40 minutes (Chen et al., 2011; Cagalinec et al., 2013). In the Su/Hx model of PAH, RV cardiomyocytes lose their regularly arranged mitochondrial ultrastructure and the mitochondria become more diffusely located throughout the cell (Shults et al., 2019). The impact of this change in mitochondrial arrangement in the PAH RV (if replicated) may have implications for bioenergetics and RVF.

Furthermore, RV fibroblasts in MCT-PAH demonstrate increased DRP1-mediated mitochondrial fission, which promotes fibroblast hyperproliferation and increases collagen deposition (Tian et al., 2018). While both Mdivi-1 and P110 treatment reduce the proliferation of RV fibroblasts, RV fibrosis appears to be P110-resistant, though Mdivi-1 retains therapeutic benefit by reducing collagen production (Tian et al., 2018). Therefore, DRP1 activation appears to play complex roles in both obstructive vascular remodeling as well as RVF through RV stiffening by fibrosis and cardiomyocyte dysfunction, making it a central player in the progression of the PAH disease phenotype.

DRP1 is upregulated and activated in most disease-relevant cells in PAH, both in animal models and patients, making it an attractive therapeutic target. However, there remain many knowledge gaps regarding the mechanisms by which DRP1 regulates cell cycle progression in vascular cells in PAH. Perhaps most notably there appear to be marked temporal changes in the expression and activity of fission and fusion mediators which require better definition. In addition, the relative contribution of the four major DRP1 binding partners in PAH remains unclear. The role of DNM2 in fission in general and in the increased mitotic fission seen in PAH requires further study. Finally, the role of peripheral *versus* midzone fission in PAH requires further study.

## 4 Conclusion and future directions

This review describes our current understanding of the various facets of acquired mitochondrial dysfunction in PAH. Rapidly accumulating evidence, from many research groups, identifies metabolic reprogramming and fusion-fission imbalance in the pathogenesis of PAH and suggests a central role for acquired mitochondrial dysfunction in this syndrome. As stewards of glucose, glutamine, and fatty acid metabolism, mitochondria produce both energy and macromolecules that regulate cellular signaling, hypertrophy, and proliferation. Their control over calcium flux affects the contractility and metabolism of PASMC. Disorders of mitochondrial fission and fusion also confer resistance to apoptosis, which underlies the obstructive, obliterative nature of the PAH vasculopathy. The obligatory nature of DRP1-mediated mitotic fission is an increasingly accepted mechanism by which mitochondria regulate the cell cycle both in PAH and cancer. In the modern era, the 10 approved vasodilator therapies for PAH improve symptoms, increase functional capacity and improve hemodynamics. However, despite these therapies, PAH remains a fundamentally life-altering condition. The role of acquired disorders of mitochondrial metabolism and dynamics in PAH merit further study and offer many promising targets for therapy, including DRP1 and its binding partners, MiD49 and MiD51. Other promising mitochondrial therapeutic targets in PAH relate to mitochondrial metabolism and calcium flux and include PKM2 and PDK, and the MCUC.

## Glossary

AKT: Protein kinase b
ASCT2: Alanine-serine-cysteine transporter 2
ATP: Adenosine triphosphate
BMPR2: Bone morphogenetic protein receptor type 2
BPTES: *N,N’*-[Thiobis(2,1-ethanediyl-1,3,4,-thiadiazole-5,2,-diyl)]bisbenzeneacetamide
**Ca**_**L**_: L-type calcium channel
**Ca**_**T**_: T-type calcium channel
CDK1: Cyclin-dependent kinase 1
cGMP: Cyclic guanosine monophosphate
COX: Cytochrome c oxidase
DA: Ductus arteriosus
DASMC: Ductus arteriosus smooth muscle cell
DNM1/2/3: Dynamin 1/2/3
DON: 6-diazo-5-oxo-L-norleucine
DRP1: Dynamin-related protein 1
ECM: Extracellular matrix
EMRE: Essential mitochondrial calcium uniporter regulator
ER: Endoplasmic reticulum
ERK: Extracellular signal-regulated kinase
ETC: Electron transport chain
**FADH**_**2**_: Flavin adenine dinucleotide
FAO: Fatty acid oxidation
FAS: Fatty acid synthase
FIS1: Fission protein 1
GED: GTPase effector domain
GLS1: Glutaminase 1
GLUT: Glucose transporter
GO: Glucose oxidation
GTP: Guanosine triphosphate
HDAC: Histone deacetylase
HIF-1α: Hypoxia-inducible factor 1-α
HK: Hexokinase
HOSS: Homeostatic oxygen-sensing system
HRE: Hypoxia response element
HPV: Hypoxic pulmonary vasoconstriction
IMM: Inner mitochondrial membrane
**IP**_**3**_ **R**: Inositol-1,4,5-triphosphate receptor
IPAH: Idiopathic pulmonary arterial hypertension
LAT1: L-type amino acid transporter 1
MAM: Mitochondria-associated membrane
MCT: Monocrotaline
MCU: Mitochondrial calcium uniporter
MCUC: Mitochondrial calcium uniporter complex
MICU1/2: Mitochondrial calcium uptake 1/2
MiD49/51: Mitochondrial dynamics proteins of 49 kDa/51 kDa
MFF: Mitochondrial fission factor
MFN1/2: Mitofusin 1/2
miR: MicroRNA
MOMP: Mitochondrial outer membrane permeabilization
mPAP: Mean pulmonary artery pressure
MPP: Mitochondrial processing peptidase
mtDNA: Mitochondrial DNA
mTOR: Mammalian target of rapamycin
NADH: Nicotinamide adenine dinucleotide
NDUFS2: NADH dehydrogenase [ubiquinone] iron-sulfur protein 2
NFAT: Nuclear factor of activated T Cells
NMDAR: N-methyl-D-aspartate receptor
NOS: Nitric oxide synthase
NRF1/2: Nuclear respiratory factor 1/2
OMM: Outer mitochondrial membrane
OPA1: Optic atrophy 1
ORAI1: Calcium release-activated calcium channel 1
OXPHOS: Oxidative phosphorylation
PA: Pulmonary arterioles
PAH: Pulmonary arterial hypertension
PAfib: Pulmonary artery fibroblast
PAEC: Pulmonary artery endothelial cell
PASMC: Pulmonary artery smooth muscle cell
PARL: Presinilin-associated rhomboid-like protease
PDH: Pyruvate dehydrogenase
PDK: Pyruvate dehydrogenase kinase
**pDRP**_**637**_: Serine 637-phosphorylated DRP1
PET: Positron emission tomography
PFK1: Phosphofructokinase 1
PGC-1α: Peroxisome proliferator-activated receptor γ coactivator 1-α
PH: Pulmonary hypertension
PINK1: PTEN-induced kinase 1
PKM2: Pyruvate kinase muscle isoform 2
PP2A: Protein phosphatase 2A
PPP: Pentose phosphate pathway
PTBP1: Polypyrimidine tract-binding protein 1
PVR: Pulmonary vascular resistance
RALA: Ras-like proto-oncogene A
RALBP1: RALA binding protein 1
ROCK: Rho kinase
ROS: Reactive oxygen species
RV: Right ventricle
RVEDP: Right ventricular end-diastolic pressure
RVF: Right ventricular failure
RVH: Right ventricular hypertrophy
SERCA: Sarco(endo)plasmic reticulum calcium ATPase
siRNA: Small interfering RNA
SOCC: Store-operated calcium channel
SOCE: Store-operated calcium entry
SOD2: Superoxide dismutase 2
SR: Sarcoplasmic reticulum
STAT3: Signal transducer and activator of transcription 3
STIM1/2: Stromal interaction molecule ½
Su/Hx-PAH: Sugen 5,416/hypoxia model of PAH
TFAM: Mitochondrial transcription factor A
TRAK1/2: Trafficking kinesin-binding protein 1/2
TRPC1: Transient receptor potential canonical 1
TRPC6: Transient receptor potential canonical 6
TTP: Tricarboxylate transport protein
VEGF: Vascular endothelial growth factor receptor
